# Astrocytic Connexin43 Channels Are Essential for Breathing Pattern Stabilization in the preBötzinger Complex

**DOI:** 10.1111/cns.70668

**Published:** 2025-11-24

**Authors:** Xue Zhao, Luo Shi, Yongqiang Chen, Hongxiao Yu, Xiaoyi Wang, Xinyi Jing, Tianjiao Deng, Ke Zhao, Xiang Zhang, Yixian Liu, Fang Yuan, Sheng Wang

**Affiliations:** ^1^ Department of Neurobiology Hebei Medical University Shijiazhuang China; ^2^ Department of Physiology Hebei Medical University Shijiazhuang China; ^3^ Hebei Key Laboratory of Brain Science and Brain‐Inspired Intelligence Shijiazhuang China

**Keywords:** astrocyte, ATP, connexin 43 channel, preBötzinger complex

## Abstract

**Objective:**

Astrocytes within the preBötzinger complex (preBötC) critically regulate respiratory rhythmogenesis and pattern formation. However, the molecular mechanisms underlying their contributions remain poorly understood. This study aims to investigate whether connexin 43 (Cx43) channels, a prominent subtype of connexin proteins expressed in preBötC astrocytes, are essential for stabilizing breathing patterns.

**Methods:**

We employed a multidisciplinary approach, integrating whole‐body plethysmography, in vivo fiber photometry, phrenic nerve discharge (PND) recordings, photostimulation, RNAscope fluorescence in situ hybridization, and RNA sequencing to elucidate the functional role of Cx43 channels in respiratory regulation.

**Results:**

Elevated activation levels of preBötC astrocytes were synchronized with specific respiratory events, including sighs and transiently augmented breathing. RNA‐sequencing analysis demonstrated that *Gja1* (encoding Cx43) was identified as the predominant connexin transcript in preBötC astrocytes. Photostimulation of preBötC astrocytes significantly increased PND frequency in anesthetized mice, an effect replicated by pharmacological blockade of Cx43 hemichannels. Conditional knockdown of astrocytic *Gja1* in the preBötC considerably increased resting breathing frequency and minute ventilation. Blockade of Cx43 hemichannels enhanced astrocytic activation and induced ATP accumulation around somatostatin‐expressing preBötC neurons (preBötC^SST^). Furthermore, Cx43 hemichannel blockade activated preBötC^SST^ neurons, an effect mediated by P2Y1 but not P2X receptors.

**Conclusion:**

We identify an astrocyte‐to‐neuron signaling cascade involving Cx43 hemichannel‐dependent ATP release, P2Y1 receptor activation on preBötC^SST^ neurons, and subsequent modulation of respiratory motor output. These findings establish Cx43 hemichannels as critical molecular determinants for stabilizing breathing patterns.

## Graphical abstract

Both pharmacological blockade and genetic knockdown of astrocytic connexin43 channels in the preBötzinger complex induce a high‐frequency breathing pattern, thereby enhancing respiratory motor output. This effect is mediated by astrocytic activation and subsequent ATP release, which activates P2Y1 receptors on somatostatin‐expressing preBötzinger complex neurons.

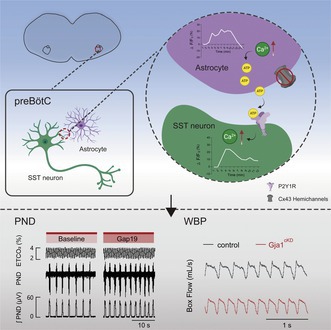

## 1 Introduction

Breathing is a fundamental physiological process essential for sustaining life. It relies on a complex neural network to orchestrate oxygen intake and carbon dioxide elimination to meet metabolic demands [1, 2, 3]. Respiratory rhythm and pattern generation are primarily governed by specialized neural circuits termed respiratory central pattern generator (rCPG). The rCPG integrates inputs from excitatory and inhibitory neurons, paracrine factors, and astrocytic signaling to shape respiratory motor output. Structural deficiencies or functional impairments within the rCPG can lead to centrally disordered breathing patterns, contributing to pathologies such as sudden infant death syndrome and opioid‐induced respiratory depression [4, 5]. The preBötzinger complex (preBötC), a key component of the rCPG, constitutes the core inspiratory rhythm‐ and pattern‐generating kernel [6, 7, 8, 9]. Emerging evidence has revealed that inspiratory rhythmogenesis primarily depends on *Dbx1*‐expressing preBötC neurons, whereas somatostatin‐expressing neurons (preBötC^SST^) contribute to shaping respiratory output [10]. Additionally, accumulated evidence highlights that dynamic interactions between neurons and astrocytes are critical for modulating the stability and adaptability of respiratory circuits [11, 12].

Astrocytes modulate synaptic transmission primarily through gliotransmitter release (e.g., ATP) in response to diverse stimuli, including hypoxic/hypercapnic challenges [13]. Specifically, astrocytes within the preBötC are regarded as regulators of respiratory rhythm‐generating circuits and determinants of exercise capacity [14]. During hypoxia, preBötC astrocytes release ATP, which acts via purinergic receptor P2Y1 on inspiratory neurons and/or glia to trigger the ventilatory increase that counteracts hypoxic respiratory depression [15]. Furthermore, photostimulation of preBötC astrocytes is sufficient to elicit sigh activity, a response blocked by purinergic receptor antagonists [16]. These findings collectively demonstrate that astrocytes exert a significant influence over both respiratory rhythm and pattern. Nevertheless, the underlying molecular mechanisms by which astrocytes shape respiratory motor output remain incompletely defined.

Connexin43 (Cx43) is the principal subunit of both gap junctions and hemichannels. Gap junctions consisting of Cx43 in astrocytes, facilitate the intercellular diffusion of ions, metabolites (e.g., glucose, lactate), and signaling molecules (e.g., ATP, glutamate) [17, 18, 19]. These Cx43 channels are essential for maintaining neural network homeostasis. Deficiencies in Cx43 channel structure or function are closely associated with neuronal hyperexcitability [20, 21], and exacerbated pathological outcomes such as ischemic stroke and epilepsy [22, 23, 24]. Despite its recognized importance in astrocytic communication and neurological disorders, the role of Cx43 channels in preBötC astrocytes remains unexplored. This study aims to address these gaps by characterizing the contribution of preBötC astrocytic Cx43 channels in the regulation of respiratory homeostasis. Our findings reveal that these channels are essential for stabilizing breathing patterns through astrocytic activation and purinergic signaling acting on preBötC^SST^ neurons.

## 2 Materials and Methods

### 2.1 Animals

SST‐IRES‐Cre mice (obtained from Jackson Laboratory, stock number 013044) and C57BL/6J mice (sourced from Charles River, catalog number #632) were utilized in this study. Both male and female mice aged 8–12 weeks and weighing 23–30 g were randomly allocated to control and experimental groups. The distribution of male and female mice across each experimental group, along with an analysis of potential sex differences in respiratory function, is detailed in Data S1 and Figure S1, respectively. Mice were housed in a 12‐h light/12‐h dark cycle (lights on at 07:00 a.m. and off at 7:00 p.m.). The housing conditions were maintained at a constant temperature of 23°C–25°C and appropriate humidity levels (40%–60%) with free access to food and water. All animal experiments were conducted in strict compliance with the Guide for the Care and Use of Laboratory Animals. The experimental protocols were approved by the Animal Care and Ethics Committee of Hebei Medical University (#Hebmu‐P2023052).

### 2.2 Viral Vectors and Stereotaxic Surgery

The viral vectors employed in this study were sourced from Brain Case (Wuhan, China) and included the following: rAAV5‐GfaABC1D‐jGCaMP7b (3.00 × 10^12^ genomic copies per mL, BC‐1341), rAAV5‐GfaABC1D‐hChR2(H134R)‐P2A‐EGFP, (5.07 × 10^12^ genomic copies per mL, BC‐2955), rAAV5‐GfaABC1D‐EGFP (5.01 × 10^12^ genomic copies per mL, BC‐0377), rAAV5‐U6‐SasgRNA(mGja1)‐CMV‐EGFP (5.03 × 10^12^ genomic copies per mL, BC‐2513), rAAV5‐U6‐SasgRNA(NC‐1)‐CMV‐EGFP (5.10 × 10^12^ genomic copies per mL, BC‐2516), rAAV5‐GfaABC1D‐SaCas9 NLS‐3 × HA (5.13 × 10^12^ genomic copies per mL, BC‐2517), rAAV9‐hSyn‐DIO‐ATP1.0 (5.02 × 10^12^ genomic copies per mL, BC‐1522), rAAV9‐EF1α‐DIO‐GCaMP6m (5.05 × 10^12^ genomic copies per mL, BC‐0087). The viral vectors were stored at −80°C to prevent repeated freeze–thaw cycles. All procedures involving the viruses were conducted under strict sterile conditions.

All mice were anesthetized intraperitoneally with pentobarbital sodium at a dose of 60 μg/g. The depth of anesthesia was assessed by the absence of corneal and digital clipping reflexes. The mice were then positioned prone on a stereotaxic apparatus (RWD, Plainview, NY, USA). A heating pad was used to maintain the body temperature at 36°C ± 1°C, and ophthalmic ointment was applied to protect the eyes. After fully exposing the injection site, a pump (Harvard Instruments, Holliston, MA, USA) was used for viral vector delivery. For pressure injection, viral solution was injected at a rate of 30 nL/min. Following injection, a diffusion period of at least 5 min was allowed before slowly withdrawing the pipette. The injection site was the bilateral preBötC (30 to 40 nL per side) according to the Mouse Brain in Stereotaxic Coordinates [25]. After that, each mouse received injections of the antibiotic ampicillin (125 mg/kg, i.p.) and ibuprofen (4 mg/kg). They were allowed to recover for 4 weeks until resuming normal activity before the next experimental measurements were made.

### 2.3 Measurement of Respiratory Function

Respiratory function in freely behaving mice was monitored using a whole‐body plethysmography (WBP) system (EMKA Technologies, France; Data Sciences International, USA), as previously reported [26]. A mass‐flow regulator was employed to supply a steady, quiet, and smooth airflow (0.5 L/min) within the WBP chamber (480 mL). Ventilatory flow signals were captured, amplified, converted into digital form, and subsequently analyzed using IOX 2.10 (EMKA Technologies) and FinePointe (DSI, USA) software. Breathing parameters were assessed over consecutive 120 s epochs (300–400 breaths) during periods of behavioral calmness and regular breathing patterns. Prior to testing, each mouse was acclimatized in the recording chamber for a minimum of 2 h.

The following respiratory parameters were measured: breathing frequency (BF, breaths/min), tidal volume (TV, μL/g), minute ventilation (MV, μL/min/g), and peak inspiratory flow (PIF, mL/s). MV was calculated as the product of BF and TV. TV, MV, and PIF values were normalized to body weight. Specific breathing patterns, including sighs and transiently augmented breathing (TAB), were also analyzed. Sighs were identified based on their characteristic large amplitude (≥ 2‐fold tidal volume) [27]. TAB events were defined as 3–10 s of consecutive breaths with their average PIF ≥ 15% increase from baseline PIF.

### 2.4 Fiber Photometry

To monitor dynamic Ca^2+^ signals of preBötC astrocytes, an AAV vector (rAAV5‐GfaABC1D‐jGCaMP7b) was unilaterally injected into the preBötC. Three weeks postinjection, an optic fiber was implanted ipsilaterally into the same region. Photometric recordings were performed using a single‐channel fiber photometry recording system (ThinkerTech, China) one week after fiber implantation to ensure adequate animal recovery. Astrocytes expressing jGCaMP7b were stimulated with laser intensities of 470 nm wavelength (30–50 μW) to elicit Ca^2+^‐dependent fluorescence signals, while a 410 nm signal (20 μW) was used to correct for movement artifacts. Light emission was captured using an sCMOS camera. Changes in Ca^2+^ signals (ΔF/F) were calculated as (F—F_0_)/F_0_, where F_0_ represents the averaged baseline fluorescence signal. Data were analyzed using MATLAB, with a significant astrocytic Ca^2+^ signal enhancement defined as a fluorescence increase of ≥ 30% above baseline.

Mice were acclimated in the WBP chamber for 120 min prior to recording. To examine the relationship between preBötC astrocytic Ca^2+^ activity and respiratory events, Ca^2+^ signals were recorded continuously for 4 h. During the recording, animals were allowed to freely explore in the WBP chamber while their breathing parameters, plethysmography traces, and Ca^2+^ activity were simultaneously monitored. A 3‐s period immediately preceding each respiratory event was designated as the baseline for analysis.

### 2.5 Phrenic Nerve Discharge (PND) Recording

The protocol was previously described [28]. Mice were anesthetized with urethane (1.3 g/kg, i.p.), with supplementary doses of 0.1 g/kg administered intravenously as required. Adequate anesthesia was confirmed by the absence of hindlimb retraction in response to toe pinch. Each mouse was positioned prone in a stereotaxic frame. Body temperature was maintained at 36°C ± 1°C using a program‐controlled heating pad. Under general anesthesia, a tracheostomy was initially conducted, followed by a bilateral vagotomy to abolish the pulmonary stretch reflex and other potential visceral reflexes. This intervention was critical in minimizing the influence of peripheral feedback mechanisms, thereby ensuring that the observed physiological outcomes could be predominantly attributed to injections of drugs into the preBötC. Following administration of the paralytic agent pancuronium (5 mg/kg body weight, i.p.), artificial ventilation with 100% O_2_ (SAR1000, CWE Inc., USA) was maintained throughout surgery to inactivate peripheral chemoreceptors. The left phrenic nerve was carefully isolated from surrounding tissues, placed on a silver bipolar electrode, and immersed in warm liquid paraffin. End‐tidal CO_2_ (ETCO_2_), an indicator of arterial blood CO_2_ partial pressure, was continuously monitored using a capnograph (MicroCapStar, CWE Inc., USA) and maintained at approximately 4% as the baseline level, with adjustments made as needed by modifying ventilator parameters. All analog data were processed using a micro1401 digitizer (Cambridge Electronic Design Ltd., UK) and analyzed offline with Spike 2 software (Cambridge Electronic Design, RRID:SCR_000903). The integrated PND was derived by rectifying and smoothing (time constant: 0.05 s) the original signal, which was sampled at 2 kHz and filtered with a 30–3000 Hz bandpass. The frequency and peak amplitude of the integrated PND (∫ PND) were used for quantitative analysis. Minute output of PND was calculated as the product of its frequency and integrated amplitude over a 1‐min period.

### 2.6 Optogenetic Stimulation

To perform photostimulation of target astrocytes, a virus encoding ChR2 was injected into the preBötC. Four weeks postviral injection, immunohistochemical experiments were carried out to validate ChR2 expression in target astrocytes. To employ in vivo photostimulation, an occipital craniotomy was carried out to expose the dorsal surface of the medulla oblongata over the preBötC. An optical fiber (200 μm in diameter) connected to a 473 nm LED source (Newdoon Inc., China) was positioned closely above the preBötC. The power of light at the end of the fiber tip was set to 10 mW in all experiments unless otherwise indicated, as measured with an optical power meter (PM20; Thorlabs, USA). Pulse widths were set to 20 ms and delivered in trains of 1–20 Hz stimulation rates based on the purpose of the experiments.

### 2.7 Confocal Optical Imaging in Brain Slices

The experimental protocol was applied as described previously [29]. In brief, adult SST‐Cre or C57BL/6J mice were anesthetized (urethane, 1.8 g/kg, i.p.) and decapitated. Transverse brainstem slices (220 μm) were prepared using a vibratome (VT1200S, Leica) in ice‐cold sucrose‐based solution (in mM: 260 sucrose, 3 KCl, 5 MgCl_2_, 1 CaCl_2_, 1.25 NaH_2_PO_4_, 26 NaHCO_3_, 10 glucose, 1 kynurenic acid; pH 7.4). The slices were incubated for 60 min in carbogen‐saturated (95% O_2_/5% CO_2_) aCSF (in mM:125 NaCl, 3 KCl, 1.2 KH_2_PO_4_, 1.2 MgSO_4_, 25 NaHCO_3_, 2 CaCl_2_·2H_2_O, 10 glucose; pH 7.4, 300 mOsm) at room temperature. Following incubation, slices were transferred to a recording chamber mounted on an upright laser‐scanning confocal microscope (LSM800, Zeiss) and continuously superfused at a flow rate of 1.5 mL/min.

For confocal imaging, SST‐Cre mice were injected with rAAV9‐EF1α‐DIO‐GCaMP6m or rAAV9‐hSyn‐DIO‐ATP1.0 into the preBötC, while C57BL/6J mice received rAAV5‐GfaABC1D‐jGCaMP7b 4 weeks prior to data collection. Target cells were visualized using the confocal microscope, and time‐series images were acquired every 0.5 s using ZEN software (Zeiss, Germany). Changes in intracellular Ca^2+^ or ATP concentrations were normalized to baseline and expressed as fold‐change in ΔF/F_0_, calculated as [(F − F_0_)/F_0_], where F_0_ represents the average background‐subtracted baseline fluorescence and F denotes the background‐subtracted peak fluorescence of each event. A significant change in astrocytic Ca^2+^ signaling was defined as ΔF/F_0_ ≥ 5% from baseline. To assess connexin hemichannel activity, Gap19 (1 μM) was bath‐applied, and tetrodotoxin (TTX, 1 μM) was co‐applied to exclude action potential‐related ATP release.

### 2.8 Single‐Cell RNA Sequencing

To characterize the transcriptional landscape of neurons in the preBötC under baseline (unstimulated) conditions, adult C57BL/6J mice (8–10 weeks old) were deeply anesthetized with urethane at a dose of 1.8 g/kg (i.p.) and then decapitated. The brainstem was quickly removed and submerged in ice‐cold cutting solution containing (in mM): 260 sucrose, 3 KCl, 2 MgCl_2_, 2 CaCl_2_, 1.25 NaH_2_PO_4_, 26 NaHCO_3_, 1 glucose, and 1 kynurenic acid, equilibrated with 95% O_2_ and 5% CO_2_ to maintain a pH of 7.3–7.4. Coronal slices (300 μm thick; bregma: approximately −6.6 mm to −7.3 mm) were sectioned using a vibratome (VT1200S; Leica Biosystems, Germany). The preBötC region was anatomically localized using established landmarks (nucleus ambiguus) under a stereomicroscope, and bilateral preBötC tissues were carefully microdissected from each slice. Tissue from 40 mice was pooled and immediately frozen in liquid nitrogen, then stored at −80°C until further processing for single‐nucleus RNA sequencing (snRNA‐seq).

Obtained brain tissues were processed for nucleus isolation, library preparation, and sequencing by Gene Denovo Biotechnology Co. Ltd. (Guangzhou, China) according to the guidelines of 10X Genomics (10X Genomics, USA). Approximately 30 mg of tissue from the preBötC of 40 mice was pooled and dissociated into a single‐nucleus suspension. The brain tissue was homogenized in ice‐cold homogenization buffer (0.25 m sucrose, 5 × 10^−3^ m CaCl_2_, 3 × 10^−3^ m MgAc_2_, 10 × 10^−3^ m Tris–HCl (pH = 8.0), 0.1 × 10^−3^ m EDTA, 1× protease inhibitor, and 1 U μL^−1^ RiboLock RNase inhibitor) with pestle strokes. Next, the homogenates were filtered through a 70 × 10^−6^ m cell strainer to collect the nuclear fraction. The nuclear fraction was mixed with an equal volume of 50% iodixanol and added on top of a 30% iodixanol solution. This solution was then centrifuged for 20 min at 10,000 g at 4°C. After the myelin layer was removed from the top of the gradient, the nuclei were collected from the 30% iodixanol interface. The nuclei were resuspended in nuclear wash buffer and resuspension buffer [0.04% bovine serum albumin, 0.2 U μL^−1^ RiboLock RNase inhibitor, 500 × 10^−3^ m mannitol, and 0.1 × 10^−3^ m phenylmethanesulfonyl fluoride (as a protease inhibitor) in PBS] and pelleted for 5 min at 500 g and 4°C. The nuclei were filtered through a 40 × 10^−6^ m cell strainer to remove cell debris and large clumps. The nuclear concentration was manually assessed using trypan blue counterstaining and a hemocytometer. Finally, the nuclear concentration was adjusted to 700–1200 nuclei μL^−1^, and the nuclei were examined with a 10× Chromium platform. Reverse transcription, cDNA amplification, and library preparation were performed based on the protocol from the manufacturer.

### 2.9 Immunohistochemistry and RNAscope‐FISH

Mice were deeply anesthetized with urethane (1.8 g/kg, i.p.) and transcardially perfused with 4% paraformaldehyde (PFA) in phosphate‐buffered saline (PBS, pH = 7.4). Whole brain tissues were collected and postfixed in 4% PFA at 4°C for 12 h. Subsequently, tissues were dehydrated through a graded series of sucrose solutions (15% and 30% in PBS) at 4°C, embedded in OCT compound, and stored at −80°C. Coronal sections (15 μm) were obtained using a freezing microtome (CM1950; Leica Microsystems, Germany).

Tissue sections were washed six times in PBS (3 min each) and incubated overnight at 4°C with primary antibodies diluted in PBS containing 5% bovine serum albumin (BSA) and 0.25% Triton X‐100. The following primary antibodies were used: rabbit anti‐GFAP (1:500, catalog #ab7260, RRID:AB_305808, Abcam), mouse anti‐NeuN (1:200, catalog #NBP1‐92693, RRID:AB_11036146, Novus Biologicals, part of Bio‐Techne), mouse anti‐Phox2b (1:200, catalog #sc‐376,997, RRID:AB_2813765, Santa Cruz Biotechnology), guinea pig anti‐cFos (1:200, catalog #226308, RRID:AB_2905595, Synaptic Systems) and chicken anti‐GFP (1:2000, catalog #ab13970, RRID:AB_300798, Abcam). Following incubation with primary antibodies, sections were washed six times in PBS (3 min each) and incubated with fluorescent secondary antibodies for 2 h at room temperature. The secondary antibodies used were donkey F(ab')2 anti‐rabbit IgG H&L (Alexa Fluor 488) preadsorbed (1:1000, catalog #ab181346, RRID:AB_2813899, Abcam), goat anti‐mouse IgG H&L (Alexa Fluor 647) preadsorbed (1:1000, catalog #ab150119, RRID:AB_2811129, Abcam), goat anti‐chicken IgY H&L (Alexa Fluor 488) (1:1000, catalog #ab150169, RRID:AB_2636803, Abcam), goat anti‐guinea pig IgG H&L (Alexa Fluor 647) preadsorbed (1:200, catalog #ab150187, RRID:AB_2827756, Abcam), and donkey anti‐rabbit IgG H&L (Alexa Fluor 647) preadsorbed (1:1000, catalog #ab150063, RRID:AB_2687541, Abcam). All washing and incubation steps were performed on a shaker at moderate speed. After a final wash in PBS (6 times, 3 min each), sections were mounted on slides using Vectashield Antifade Mounting Medium (Vector Laboratories, Burlingame, CA, USA) for visualization.

Fluorescence in situ hybridization (FISH) was performed using the RNAscope Multiplex Fluorescent Reagent Kit V2 (ACDbio) according to the manufacturer's instructions. Briefly, brain tissues embedded in OCT were sectioned to a thickness of 15 μm. To combine immunohistochemistry with RNAscope, the protocol was followed up to the hydrogen peroxide wash step. Sections were then washed in PBS and incubated overnight at 4°C with primary antibodies diluted in Co‐Detection Antibody Diluent (ACDBio). After washing in PBS, sections were treated with Protease Plus, and the RNAscope protocol was resumed. Sections were washed in Wash Buffer Reagents (ACDBio) and incubated with a goat polyclonal secondary antibody diluted in Co‐Detection Antibody Diluent for 30 min at room temperature. Finally, sections were washed in PBS and mounted with Fluoromount‐G (SouthernBiotech). Images were acquired using a laser scanning confocal microscope (LSM 800; Carl Zeiss, Jena, Germany) and processed using ZEN software (Carl Zeiss). Cells were manually counted in confocal images. The following RNAscope probe was used: *Gja1*‐C1 (catalog #486191‐C1, Advanced Cell Diagnostics).

### 2.10 Quantitative Real‐Time PCR (qPCR) Analysis

Mice were deeply anesthetized (urethane, 1.8 g/kg, i.p.). Brainstem tissues were rapidly dissected and sectioned into 200–300 μm coronal slices using a vibratome (VT1200S, Leica Biosystems). The preBötC was dissected under stereomicroscopic guidance following anatomical landmarks. Total RNA extraction was performed using the Eastep Super Total RNA Extraction Kit (Promega, Cat# LS1040) following the manufacturer's protocol. cDNA synthesis was conducted with 1 μg RNA using HiScript III RT SuperMix (Vazyme). The qPCR reactions were prepared in technical triplicates using ChamQ Universal SYBR qPCR Master Mix (Vazyme, Cat# R323‐01) on a CFX‐96 Real‐Time System (Bio‐Rad). The thermal cycling protocol comprised: initial denaturation at 95°C for 30 s, followed by 40 cycles of 95°C for 10 s and 60°C for 30 s, with a final melt curve stage (95°C for 15 s, 60°C for 1 min, 95°C for 15 s). Primer sequences were retrieved from PrimerBank and synthesized by Sangon Biotech (Shanghai, China). The sequences are listed below (5′ to 3′): GAPDH (F: AGGTCGGTGTGAACGGATTTG, R: TGTAGACCATGTAGTTGAGGTCA; 123 bp; PrimerBank ID: 6679937a1), Gja1 (F: ACAGCGGTTGAGTCAGCTTG, R: GAGAGATGGGG AAGGACTTGT; 106 bp; PrimerBank ID: 33286888a1), GFAP (F: CGGAGACGCATCACCTCTG, R: AGGGAGTGGAGGAGTCATTCG; 126 bp; PrimerBank ID: 30692526a1) and Gjb6 (F: ACCAGCATAGGGAAGGTGTG, R: TGCAGAGTGTTGCAGACAAAG; 119 bp; PrimerBank ID: 6680017a1). Relative mRNA expression was calculated using the 2^−∆∆Ct^ method with GAPDH normalization. Data were normalized to wild‐type (WT) control mice unless specified.

### 2.11 Preparation and Administration of Pharmacological Agents

Carbenoxolone disodium (CBX, a gap junction blocker; catalog# 3096, Tocris Bioscience) was diluted in sterile saline to a concentration of 100 μM. For in vivo administration, 80 nL (per injection) of the CBX stock solution was injected into the preBötC (coordinates: anteroposterior, −6.85 mm; mediolateral, ±1.38 mm; dorsoventral, −5.97 mm). Gap19 (a selective Cx43 hemichannel blocker; catalog# 5353, Tocris Bioscience) was diluted in sterile saline to a concentration of 250 μM. For in vivo use, 80 nL (per injection) of the Gap19 solution was injected into the preBötC. For in vitro use, Gap19 was diluted with artificial cerebrospinal fluid (aCSF) to a final concentration of 1 μM and bath‐applied in the perfusion chamber. PPADS tetrasodium salt (a nonselective purinergic receptor P2 antagonist; catalog# 0625, Tocris Bioscience) was diluted in aCSF to 1 μM in the perfusion chamber. MRS2365 (a selective purinergic receptor P2Y1 agonist; catalog# 2157, Tocris Bioscience) was diluted in saline to 100 μM and injected in the preBötC (80 nL per injection). MRS2279 (a selective purinergic receptor P2Y1 antagonist; catalog# 2158, Tocris Bioscience) was diluted in saline to 100 μM and injected in the preBötC (80 nL per injection). BzATP (a P2X agonist; catalog# HY‐136254, MedChemExpress) was diluted in saline to 100 μM and injected in the preBötC (80 nL per injection). To verify the acute injection site within the preBötC, Green Retrobeads IX (Lumafluor) were microinjected into the preBötC. Brain slices were then prepared, and the injection site was confirmed under a fluorescence microscope. All drug concentrations and administration protocols used in this study were based on previously established methodologies [30, 31, 32, 33, 34, 35].

### 2.12 Statistics

Statistical analyses were performed using Prism 9.0.0 (GraphPad), ImageJ, ZEN (v3.4), and Spike2 (v8.0). For paired comparisons, Student's t‐test was applied for normally distributed data, while the Wilcoxon signed‐rank test was used for nonparametric data following Shapiro–Wilk normality verification. Independent group comparisons were conducted using Student's t‐test for parametric data or the Mann–Whitney U test for nonparametric data, with Welch's correction applied in cases of unequal variances. Single‐factor multigroup comparisons were analyzed using one‐way ANOVA, followed by Dunnett's multiple comparisons test, Tukey's multiple comparisons test, or Dunn's multiple comparisons test. For assessing two‐factor interactions, two‐way ANOVA was employed, accompanied by Bonferroni's multiple comparisons test. Data are presented as mean ± SEM, and statistical significance was defined as *p* < 0.05.

## 3 Results

### 3.1 Respiratory Changes Are Associated With Astrocytic Activation in the preBötC

**FIGURE 1:**
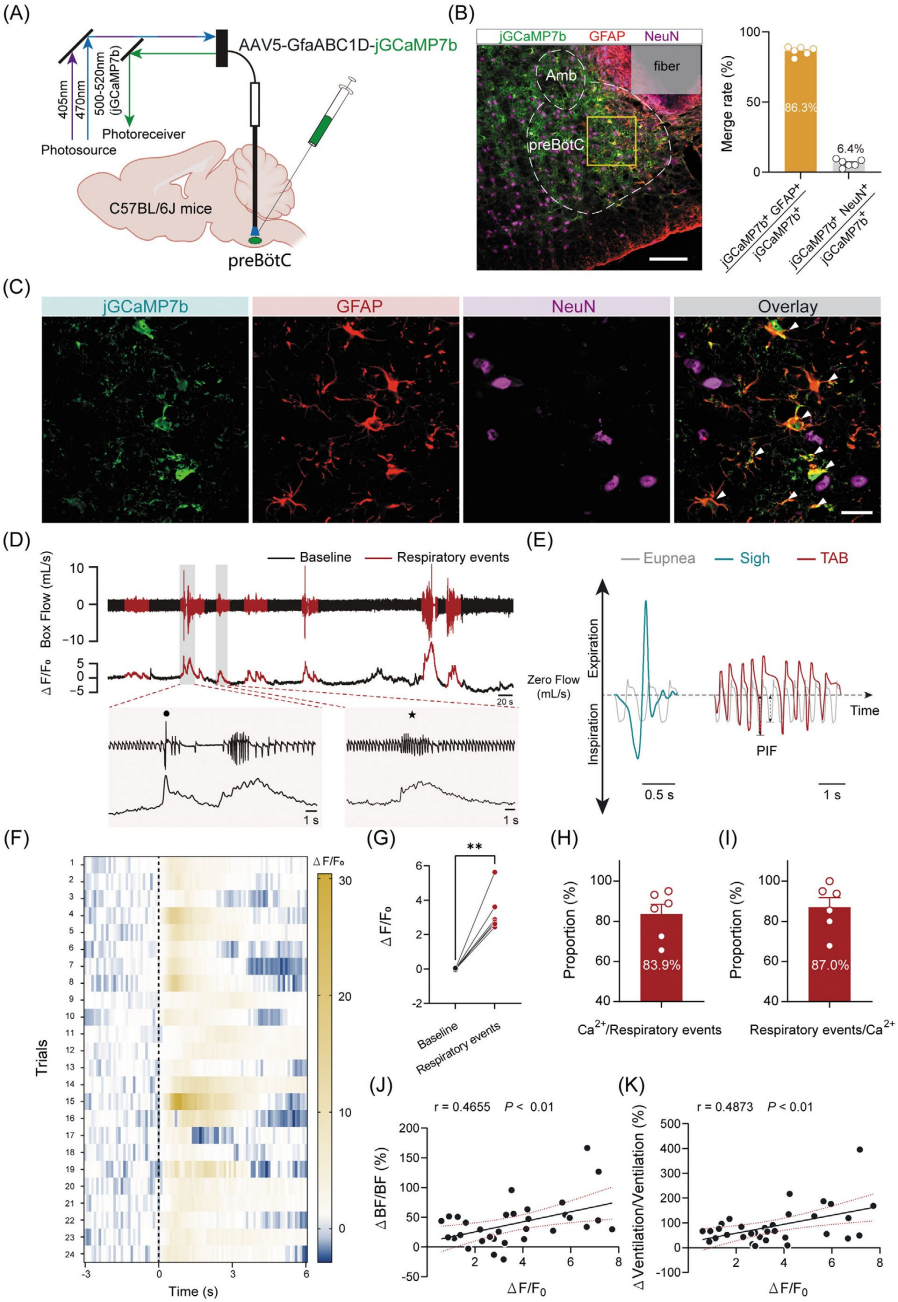
Positive correlation between preBötC astrocytic activation and respiratory events. (A) Schematic representation of the viral injection strategy and in vivo fiber photometry setup in C57BL/6J mice. (B) Validation of jGCaMP7b expression in preBötC astrocytes. *Left*: Representative photomicrograph demonstrating colocalization of jGCaMP7b (green) and GFAP (red), but not NeuN (pink), in the preBötC (coronal section). Scale bar: 100 μm. *Right*: Quantitative analysis of jGCaMP7b expression specificity. (C) Magnified views of the squared region indicated in (B). White arrowheads indicate coexpression of jGCaMP7b and GFAP. Scale bar: 20 μm. (D) Typical traces of respiratory waveforms recorded by WBP in freely behaving mice, alongside changes in astrocytic Ca^2+^ signals. *Top to bottom*: Raw respiratory waveforms, astrocytic Ca^2+^ activity and enlarged views. Black star and dot mark TAB events and sigh, respectively. (E) Enlarged views of respiratory events: Eupnea (gray), sigh (blue) and TAB (red) waveforms. (F) Heatmap depicting dynamic Ca^2+^ signals. Data were collected from four randomly selected respiratory events per mouse (*n* = 6 mice). (G) Quantitative analysis of the average Ca^2+^ signal across four respiratory events per mouse (*n* = 6 mice). (H) Proportion of elevated Ca^2+^ signal events relative to the total number of respiratory events during a 1‐h recording session. (I) Proportion of respiratory events relative to the total number of elevated Ca^2+^ signal events during a 1‐h recording session. (J) Correlation analysis of ΔBF/BF_0_ and ΔF/F_0_, with a 95% confidence interval of 0.1554 to 0.6973. (K) Correlation analysis of ΔVentilation/Ventilation and ΔF/F_0_, with a 95% confidence interval of 0.1729 to 0.7115. Sample sizes: *n* = 24 trials from 6 mice (F), *n* = 6 mice (G–I). Significance levels: **p < 0.0001 by two‐tailed paired *t* test (G), Pearson correlation coefficient (J–K). Abbreviations: Amb, nucleus ambiguus; WBP, whole body plethysmography; TAB, transiently augmented breathing.

Astrocytes in the preBötC are critical components of respiratory homeostasis networks. However, little is known regarding the temporal coupling between preBötC astrocytes and respiratory events. To address this, we employed in vivo fiber photometry and WBP recordings to assess this coupling between astrocytic activation levels and respiratory output changes in freely behaving mice. Astrocytic activation is generally indicated by changes in intracellular Ca^2+^ signaling. To monitor astrocytic Ca^2+^ signaling, we expressed the Ca^2+^ indicator jGCaMP7b specifically in astrocytes by injecting AAV5‐GfaABC1D‐jGCaMP7b into the preBötC of C57BL/6J mice (Figure 1A). Four weeks postinjection, immunohistochemical verification confirmed successful viral expression and correct placement of the optical fiber (Figure 1B). Mice with off‐target injection or misaligned fibers were excluded from subsequent analysis. Quantitative cell counting revealed that 86.3% of GCaMP7b^+^ cells (*n* = 387/446 cells from 6 mice) were GFAP^+^, while 6.4% were NeuN^+^ and 7.3% were of unknown identity, demonstrating the high specificity of GfaABC1D promoter‐driven GCaMP expression (Figure 1B,C). Note that 6.4% of GCaMP7b^+^ cells were also NeuN^+^. While the GfaABC1D promoter is strongly astrocyte‐preferential, it is not absolutely specific. Minor off‐target expression in neurons has been reported previously [36, 37].

Astrocytic Ca^2+^ signals and breathing events were assessed during behavioral quiescence. Although there were no discernible changes in astrocytic Ca^2+^ signaling with respect to the baseline respiratory rhythm and pattern, enhanced Ca^2+^ signals were observed synchronously with specific respiratory events, including sigh and TAB events (Figure 1D). Sighs were identified as a large amplitude inspiration followed by deep expiration, and the TAB event was considered a respiratory event lasting 3–10 s with an average PIF exceeding the baseline by ≥ 15% (Figure 1E–G). Quantitative analysis over a 1‐h period revealed that approximately 83.6% of sigh and TAB events were accompanied by enhanced Ca^2+^ signals (*n* = 6 mice; enhanced Ca^2+^ signals: 28 ± 4 events/h, respiratory events: 34 ± 4 events/h, Figure 1H), while 87.0% of enhanced Ca^2+^ signals were temporally aligned with specific respiratory events (*n* = 6 mice; respiratory events: 28 ± 4 events/h; enhanced Ca^2+^ signals: 33 ± 5 events/h, Figure 1I). Additionally, Pearson correlation analysis further demonstrated a positive relationship between the change in astrocytic Ca^2+^ signals and respiratory events, as indexed by changes in BF (Figure 1J) and ventilation (Figure 1K). These findings reveal a temporal coupling between preBötC astrocytic activation and respiratory changes, suggesting a potential role for these astrocytes in the regulation of breathing patterns.

### 3.2 Photostimulation of preBötC Astrocytes Potentiates Respiratory Drive

**FIGURE 2:**
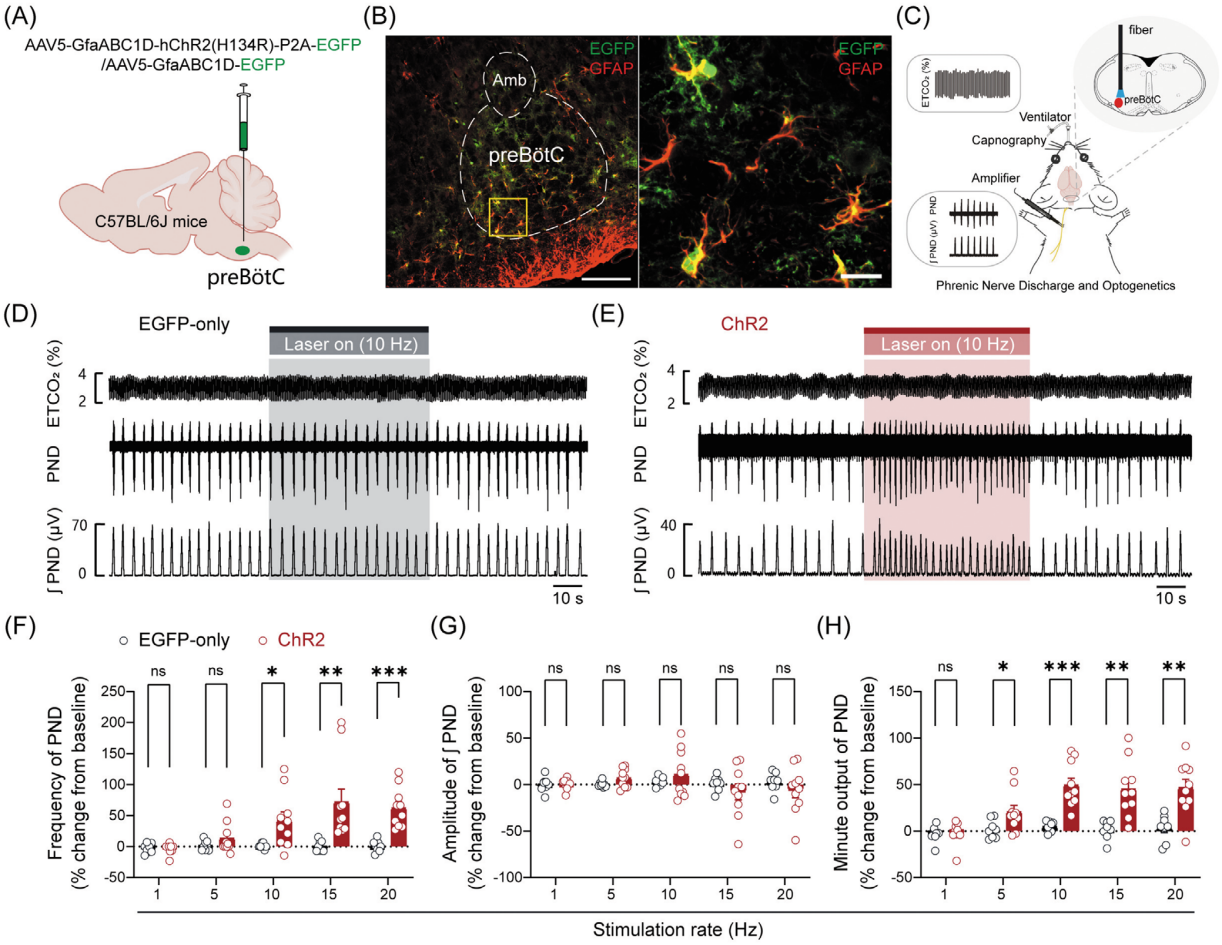
Photostimulation of preBötC astrocytes potentiates respiratory drive. (A) Schematic of the optogenetic strategy. (B) Immunohistochemical identification of ChR2‐EGFP (green) coexpressed with GFAP (red) in the preBötC. The right confocal image is an enlarged view derived from the squared region of the left image. Scale bars: 100 μm (left), 20 μm (right). (C) Schematic of the experimental setup for PND recordings in bilaterally vagotomized, mechanically ventilated, anesthetized C57BL/6J mice. (D and E) Representative traces of photostimulation (wavelength: 473 nm; rate: 10 Hz; pulse width: 20 ms) effect on PND activity. Top to bottom: ETCO_2_; raw PND traces; integrated PND derived from rectification and smoothing (time constant: 0.05 s). (F–H) Quantification of normalized PND frequency, amplitude, and minute output. Sample sizes: *n* = 8 mice for EGFP‐only (F–H), *n* = 10 mice for ChR2 (F–H). Significance levels: *p < 0.05, **p < 0.01, ***p < 0.001 by two‐tailed unpaired *t* test (10, 15, 20 Hz in F; G; 5, 10, 15, 20 Hz in H), Mann–Whitney test (1, 5 Hz in F; 1 Hz in H). ns, not significant. Abbreviations: PND, phrenic nerve discharge.

To elucidate the contribution of preBötC astrocytes to respiratory drive, we employed photostimulation and PND recordings in bilaterally vagotomized, mechanically ventilated, anesthetized mice. Initially, a viral vector encoding ChR2 (AAV5‐GfaABC1D‐hChR2‐P2A‐EGFP) or a control virus lacking ChR2 (AAV5‐GfaABC1D‐EGFP) was injected into the preBötC of C57BL/6J mice, respectively (Figure 2A). Four weeks postinjection, histomolecular validation confirmed viral targeting and successful ChR2‐EGFP expression in astrocytes (Figure 2B). Next, to assess astrocytic activation in response to optogenetic stimulation of the preBötC, we employed immunohistochemical assays to quantify cFos expression, a marker of cellular activity, following programmed photostimulation (Figure S2A,B). Given that preBötC neurons extend axonal projections to the contralateral preBötC to establish synaptic connections [38, 39], we quantified cFos^+^GFAP^+^ cells bilaterally within the preBötC. As illustrated in Figure S2C–E, the number of cFos^+^ GFAP^+^ cells was significantly elevated in mice expressing ChR2 compared to those expressing EGFP alone, both in the ipsilateral (illuminated, Figure S2C) and contralateral (nonilluminated, Figure S2D) sides of the preBötC. To further investigate whether preBötC neuronal activation might be secondary to astrocytic activation, we also quantified cFos^+^NeuN^+^ cells in both hemispheres of the preBötC. As demonstrated in Figure S2F–H, the number of cFos^+^NeuN^+^ cells was markedly increased in ChR2‐expressing mice relative to EGFP controls, both ipsilaterally (Figure S2F) and contralaterally (Figure S2G). These findings collectively demonstrate that photostimulation of the preBötC effectively induces astrocytic activation, which is accompanied by bilateral neuronal activation.

Following the validation of photostimulation‐induced astrocytic activation, we proceeded to investigate the impact of this activation effect on PND in anesthetized mice. Animals were supplied with 100% O_2_ to minimize peripheral chemoreceptor influence, and ETCO_2_ was maintained at a constant level (4%) throughout the experiment (Figure 2C). Photostimulation (473 nm laser, 60 s, 10 mW, 20 ms pulse width) of preBötC astrocytes at varying frequencies (1, 5, 10, 15, and 20 Hz) induced no significant changes in PND activity in control mice (Figure 2D,F–H). In ChR2‐expressing mice, photostimulation at 1 and 5 Hz did not alter PND frequency and amplitude. However, photostimulation at frequencies ≥ 10 Hz reliably induced a remarkable increase in PND frequency, while amplitude remained unchanged (Figure 2E–G). Additionally, the minute output of integrated PND, which reflects changes in MV, significantly increased following photostimulation (Figure 2H). These findings demonstrate that stimulation of preBötC astrocytes augments central respiratory drive, highlighting their critical role in regulating respiratory function.

### 3.3 Expression of Cx43 Channels in preBötC Astrocytes

**FIGURE 3:**
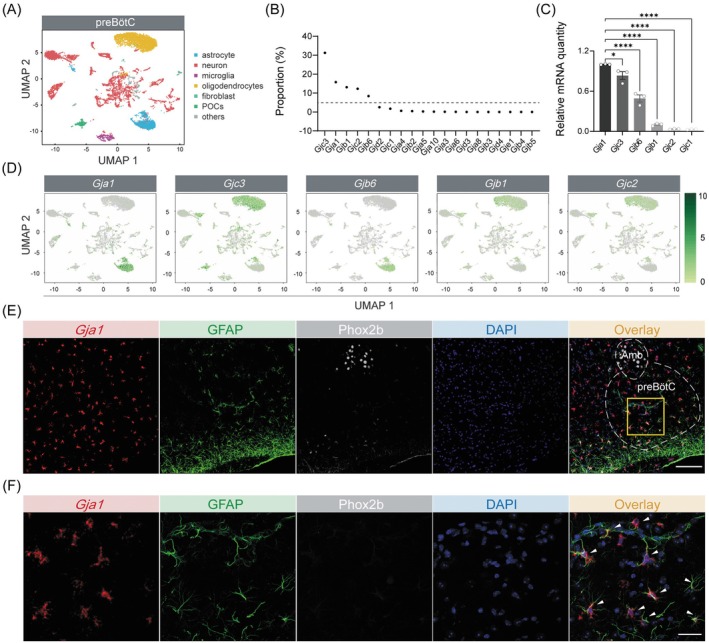
*Gja1* expression in preBötC astrocytes. (A) Single‐cell RNA sequencing analysis reveals the UMAP plot depicting the distinct cell types within the preBötC. (B) The proportional distribution of cells expressing specific connexin isoform genes across all clusters of preBötC cells. (C) qPCR analysis demonstrating the expression levels of genes encoding connexin channels in preBötC astrocytes. (D) The UMAP plot illustrating clustering of *Gja1*, *Gjc3*, *Gjb6, Gjb1*, and *Gjc2*. (E) RNAscope‐FISH and immunohistochemical analyses demonstrating the colocalization of *Gja1* mRNA (red) and GFAP protein (green) within the preBötC of C57BL/6J mice. Phox2b (gray) serves as a biomarker to label the Amb. The images were taken at a bregma level of −6.95 mm. Scale bar: 100 μm. (F) Enlarged views of the region highlighted by the yellow box in Panel E, displaying *Gja1* mRNA (red), GFAP protein (green), Phox2b (gray), and the nuclear counterstain DAPI (blue). White arrowheads indicate triple labeling for *Gja1*, GFAP, and DAPI, confirming the presence of *Gja1* mRNA in astrocytes. Scale bar: 50 μm. Sample sizes: *n* = 3 mice (C). Significance levels: *p < 0.05, ****p < 0.0001 by one‐way ANOVA with Dunnett's multiple comparisons test (C).

Although astrocytes are known to play a pivotal role in respiratory control, the molecular mechanisms underlying their function remain poorly understood. Gap junctions and hemichannels, formed by connexin proteins, are critical for maintaining brain homeostasis, with gap junctions enabling astrocytes to establish functional networks essential for homeostatic regulation. To identify the specific connexin subtypes expressed in the preBötC, we employed a single‐cell RNA‐sequencing technology on preBötC tissue from C57BL/6J mice. Uniform manifold approximation and projection (UMAP) analysis delineated distinct cell populations (Figure 3A), with astrocytes emerging as an independent cluster. Analysis of connexin gene expression revealed that *Gjc3, Gja1, Gjb1, Gjc2*, and *Gjb6* were the most abundant isoforms in the preBötC (Figure 3B), followed by further validation by qPCR analysis (Figure 3C). UMAP analysis highlighted heterogeneous expression patterns of connexin genes across cell types. Notably, *Gjc3*, *Gjb1*, and *Gjc2* were predominantly expressed in oligodendrocytes, whereas *Gja1* (encoding Cx43) and *Gjb6* (encoding connexin 30) were primarily expressed in astrocytes, with *Gja1* seemingly serving as the most abundant isoform in astrocyte‐enriched clusters (Figure 3C,D). These findings were further corroborated by RNAscope‐FISH and immunohistochemical analyses, which revealed high expression of *Gja1* mRNA colocalized highly with GFAP^+^ astrocytic processes in the preBötC (Figure 3E,F).

### 3.4 Pharmacological Blockade of preBötC Astrocytic Cx43 Channels Potentiates Central Respiratory Drive

**FIGURE 4:**
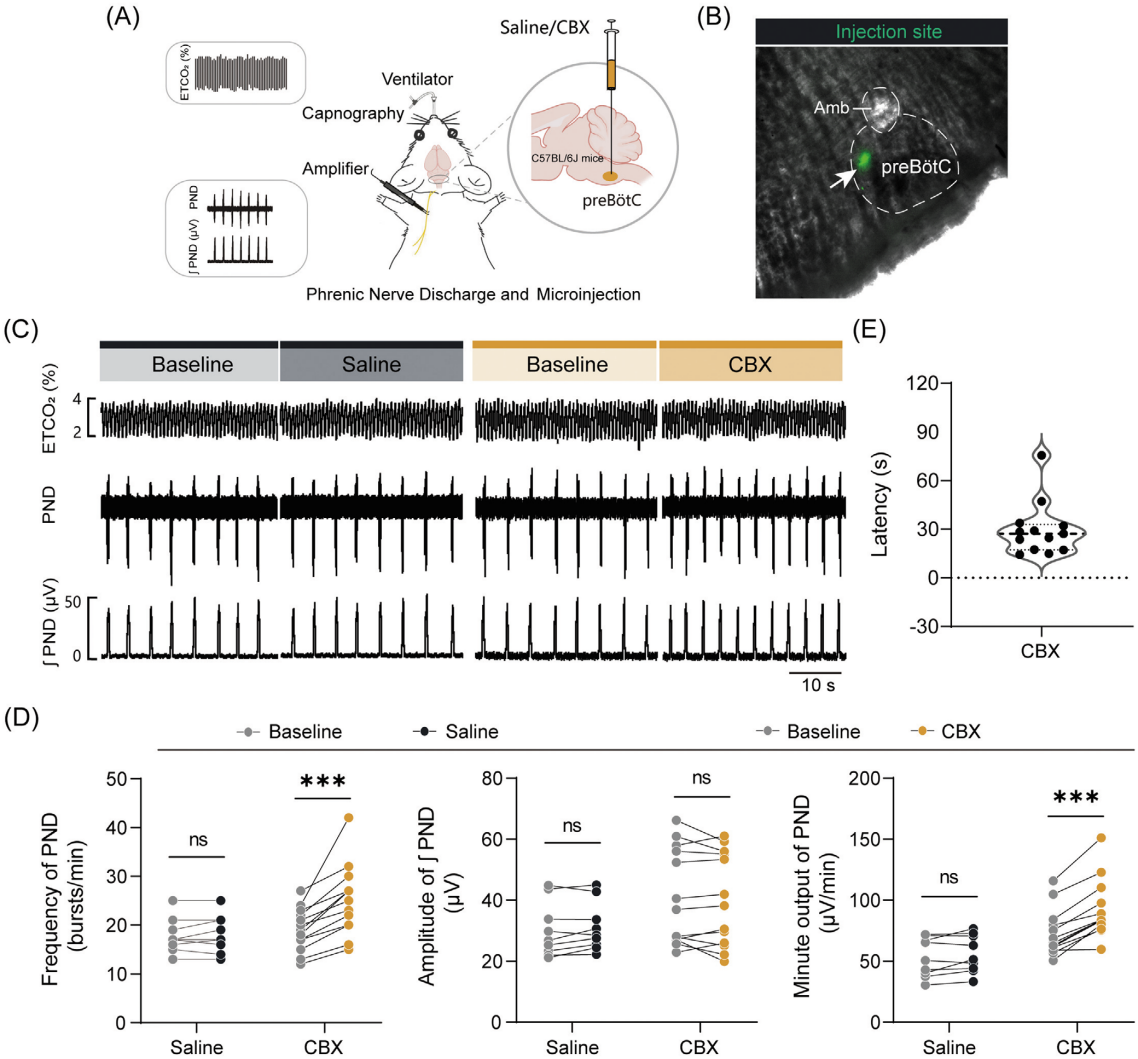
Blockade of gap junctions in the preBötC potentiates respiratory drive. (A) Schematic of PND recordings and microinjection in anesthetized C57BL/6J mice. (B) Differential interference contrast (DIC) image of a microinjection site, marked by fluorescent microspheres (green, white arrowhead). (C) Representative traces illustrating the effects of saline and carbenoxolone (CBX, 100 μM, 80 nL per injection) on PND activity. (D) Quantitative analysis of PND activity. (E) Quantification of the response latency following CBX injection. Sample sizes: *n* = 9 mice for saline (D), *n* = 13 mice for CBX (D and E). Significance levels: ***p < 0.001 by two‐tailed paired *t* test (D), Wilcoxon matched‐pairs signed rank test (minute output for CBX in D).

To elucidate the functional role of astrocytic Cx43 channels within the preBötC in respiratory regulation, we employed a pharmacological blockade strategy and evaluated its effects on central respiratory drive, which was monitored in anesthetized C57BL/6J mice using established protocols. Initially, either saline or the nonspecific gap junction antagonist CBX (100 μM, 80 nL per injection) was unilaterally microinjected into the preBötC (Figure 4A). The precise localization of the injection site was confirmed using fluorescent microbeads (Figure 4B). Administration of CBX elicited a significant increase in PND frequency (19.1 ± 1.2 to 25.1 ± 2.0 breaths/min, baseline vs. CBX, *n* = 9 mice for saline, *n* = 13 mice for CBX, *p* < 0.001), while leaving the amplitude unchanged, thereby enhancing the minute output of PND. In contrast, injection of an equivalent volume of saline produced no significant effects (Figure 4C,D). The mean latency‐to‐onset for the CBX‐induced effect was approximately 29.6 s (Figure 4E).

**FIGURE 5:**
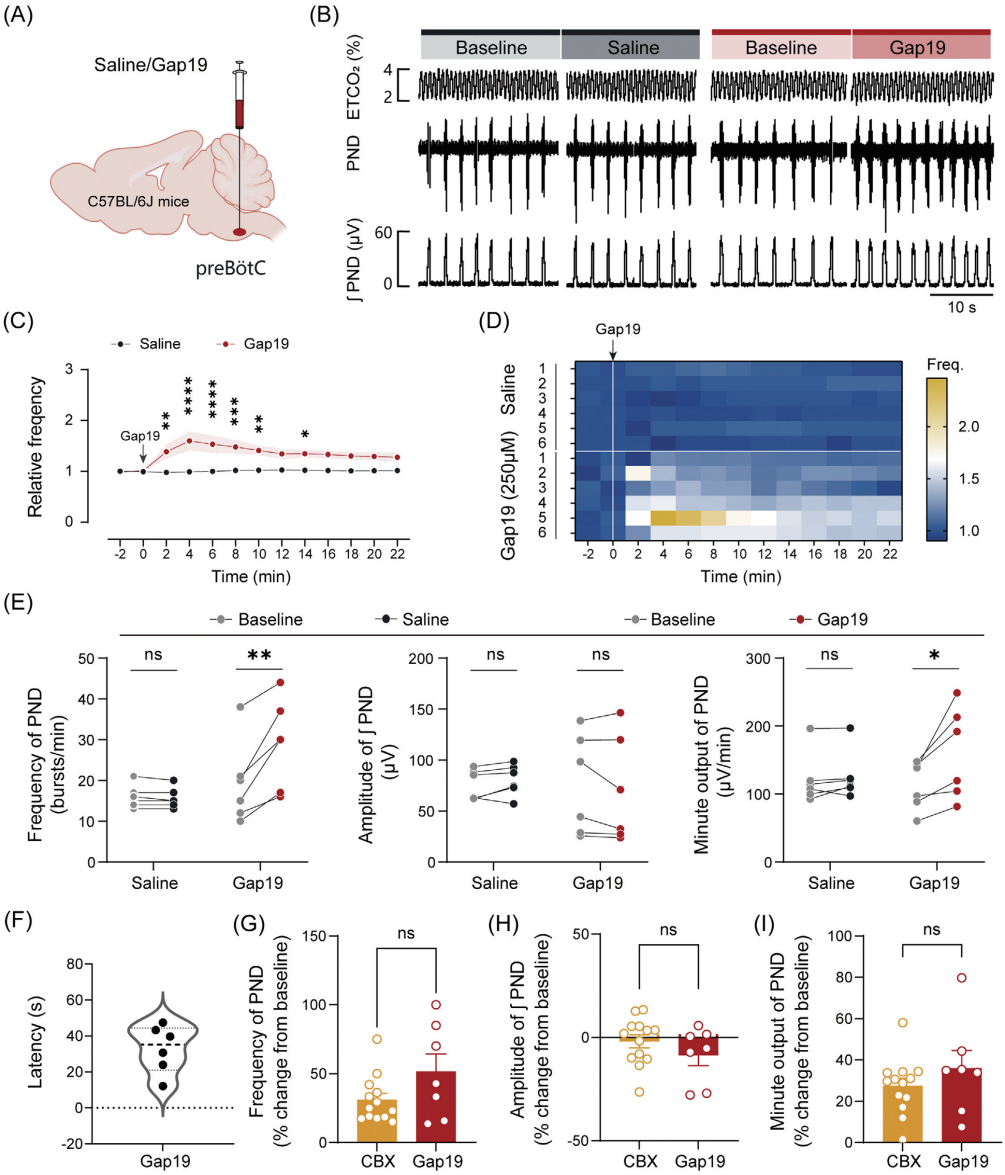
Blockade of Cx43 hemichannels in the preBötC potentiates respiratory drive. (A) Schematic of the drug injection in C57BL/6J mice. (B) Representative traces showing that the injection of Gap19 (250 μM, 80 nL per injection), but not an equivalent volume of saline, increased PND activity. (C) Time course of normalized average PND frequency in response to saline and Gap19 administration in the preBötC. (D) Heatmap illustrating the time‐course changes in PND frequency for individual mice during saline and Gap19 administration. (E) Quantitative analysis of PND activity following Gap19 administration. (F) Quantification of the response latency following Gap19 injection. (G–I) Quantification of normalized PND frequency, amplitude, and minute output in response to the injection of either CBX or Gap19 in the preBötC. Sample sizes: *n* = 6 mice in each group (C–E), *n* = 6 mice (F), *n* = 13 mice for CBX (G–I), *n* = 7 mice for Gap19 (G–I). Significance levels: *p < 0.05, **p < 0.01, ***p < 0.001, ****p < 0.0001 by two‐way ANOVA with Bonferroni's multiple comparisons test (C), two‐tailed paired t test (E), two‐tailed unpaired *t* test (G–I). ns, not significant.

To further delineate the role of astrocytic Cx43 channels in respiratory control, we administered Gap19 (250 μM, 80 nL per injection), a selective Cx43 hemichannel blocker, into the preBötC of C57BL/6J mice (Figure 5A). PND frequency exhibited a peak response approximately 4 min postinjection, followed by a gradual decline, returning to baseline levels by 15 min (Figure 5B,C). Temporal dynamics were further visualized using a frequency heatmap (Figure 5D). Gap19 significantly increased PND frequency (19.3 ± 4.1 to 29.0 ± 4.5 breaths/min, baseline vs. Gap19, *n* = 6 mice in each group, *p* < 0.01) and minute output, while leaving the amplitude unchanged (Figure 5E). In contrast, saline injections elicited no significant alterations (Figure 5C–E). The mean latency‐to‐onset for the Gap19‐induced effect was ~32.9 s (Figure 5F). Normalized data demonstrated comparable PND modulation by Gap19 and CBX (Figure 5G–I), indicating that Cx43 hemichannels represent a principal astrocytic molecule regulating respiratory output in the preBötC. This aligns with single‐cell RNA sequencing confirming *Gja1* as the predominant connexin transcript in preBötC astrocytes.

### 3.5 Impact of Cx43 Channel Knockdown in preBötC Astrocytes on Ventilation

**FIGURE 6:**
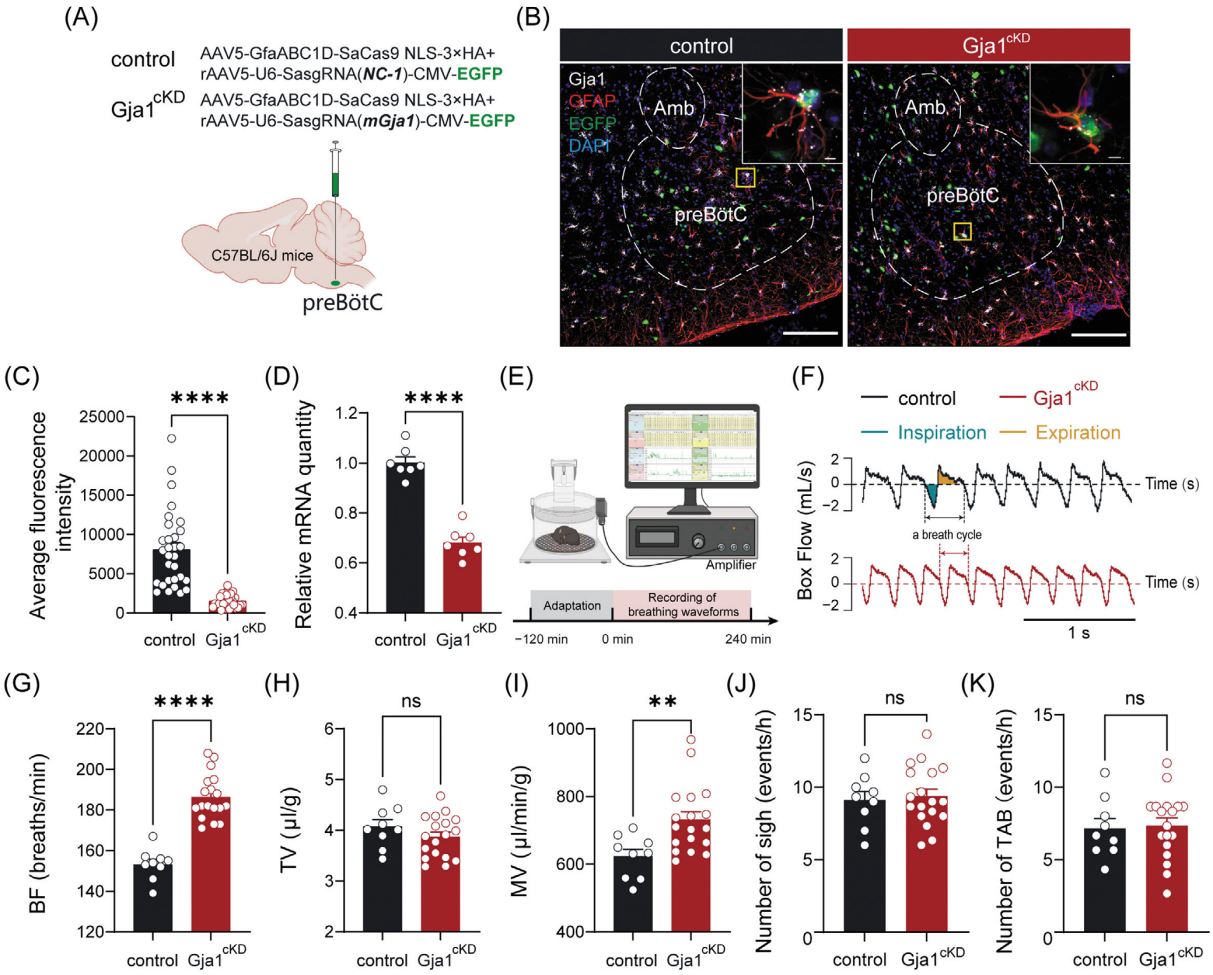
Knockdown of preBötC astrocytic Cx43 channels enhances respiratory output. (A) Schematic of the viral injection strategy. (B) RNAscope‐FISH and immunohistochemical identification of *Gja1* (encoding Cx43 channel) mRNA levels in Gja1^cKD^ mice (right) relative to controls (left). Scale bar: 100 μm. The inserts indicate enlarged views derived from the squared regions (Scale bar: 5 μm). (C) Average fluorescence intensity (normalized to area) was markedly reduced following knockdown of *Gja1*. (D) Quantitative analysis using qPCR demonstrates that expression levels of *Gja1* mRNA in Gja1^cKD^ mice were remarkably downregulated compared to control mice. (E) Schematic of the experimental setup and procedure of WBP. (F) Typical traces of original respiratory waveforms collected from Gja1^cKD^ mice and controls. Schematic diagram depicting a breath cycle composed of inspiratory (blue area) and expiratory (yellow area) phases. (G–I) Quantitative analysis of BF, TV, and MV between two groups. (J and K) Quantitative analysis of sighs and TAB events over a 1‐h period. Sample sizes: *n* = 30 cells from 3 mice (C), *n* = 7 mice in each group (D), *n* = 9 mice in control group (G–K), *n* = 18 mice in Gja1^cKD^ group (G–K). Significance levels: **p < 0.01, ****p < 0.0001 by Mann–Whitney test (C), two‐tailed unpaired *t*‐test (D and G–K). ns, not significant.

To investigate the role of astrocytic Cx43 channels in the preBötC in regulating ventilatory responses, we employed an astrocyte‐specific knockdown approach targeting *Gja1*. This was achieved by bilaterally injecting a combination of AAV5‐GfaABC1D‐SaCas9‐NLS‐3 × HA and AAV5‐U6‐sgRNA(mGja1)‐CMV‐EGFP into the preBötC of C57BL/6J mice (hereafter referred to as Gja1^cKD^ mice). Control mice received injections of a 1:1 mixture of AAV5‐U6‐sgRNA (NC‐1)‐CMV‐EGFP and AAV5‐GfaABC1D‐SaCas9‐NLS‐3 × HA (Figure 6A). Four weeks postinjection, RNAscope‐FISH combined with immunohistochemistry confirmed that EGFP expression was predominantly restricted to GFAP^+^ astrocytes (Figure 6B). Quantitative analyses revealed a significant 76.1% reduction in *Gja1* mRNA fluorescence intensity (Figure 6C) and a corresponding 31.9% decrease in *Gja1* transcript levels as measured by qPCR in the preBötC of Gja1^cKD^ mice compared to controls (Figure 6D). These findings validate the successful and specific knockdown of *Gja1* in astrocytes, providing a robust model to assess the functional consequences of astrocytic Cx43 channels disruption on ventilatory control.

To evaluate the impact of astrocytic Cx43 channel knockdown on ventilatory control, we utilized WBP to measure respiratory parameters in freely behaving mice during periods of behavioral quiescence (Figure 6E,F). Quantitative analysis of 3‐h recordings revealed a significant increase in BF (153.2 ± 2.6 vs. 186.3 ± 2.6 breaths/min, control vs. Gja1^cKD^; *p* < 0.0001; Figure 6G) and MV (623.2 ± 21.1 vs. 731.3 ± 23.4 μL/min/g, control vs. Gja1^cKD^; *p* < 0.01; Figure 6I) in Gja1^cKD^ mice (*n* = 18 mice) relative to controls (*n* = 9 mice), while TV remained unchanged (Figure 6H). The frequency of sighs and TAB events was unaffected by the knockdown (Figure 6J,K). These results collectively demonstrate that astrocytic Cx43 channel knockdown induces a high‐frequency breathing phenotype, underscoring their critical role in stabilizing respiratory patterns.

Previous studies have suggested that connexin 30 (Cx30) channels may be upregulated in astrocytes as a compensatory mechanism following Cx43 deletion or functional impairment [40, 41]. To explore this possibility in our model, we examined Cx30 expression in the preBötC after Cx43 knockdown. No significant increase in Cx30 expression was observed (Figure S3), indicating that functional compensation by Cx30 is unlikely to play a major role in the observed respiratory phenotype. These findings further emphasize the essential contribution of astrocytic Cx43 channels to the regulation of respiratory rhythm and pattern generation.

### 3.6 Blockade of Cx43 Hemichannels Enhances preBötC Astrocytic Activation Levels

**FIGURE 7:**
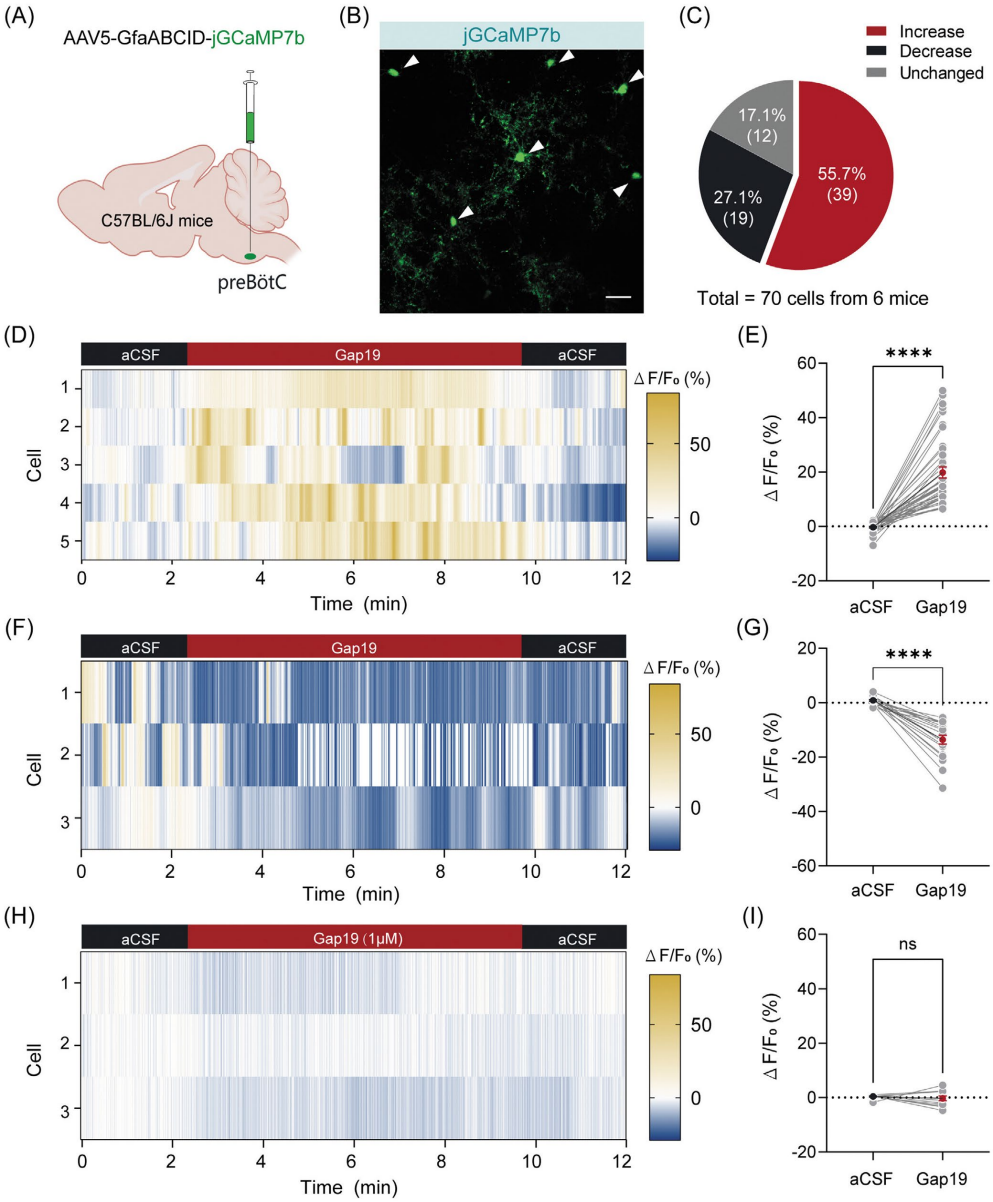
Blockade of preBötC astrocytic Cx43 hemichannels modulates Ca^2+^ signaling. (A) Schematic illustration of the viral injection strategy employed to target astrocytes in the preBötC. (B) Representative confocal image of jGCaMP7b‐expressing astrocytes (green, white arrowheads) in a slice from a C57BL/6J mouse. Scale bar, 20 μm. (C) Responsive patterns of astrocytic Ca^2+^ signals in response to the blockade of Cx43 hemichannels by bath application of Gap19. (D–I) Heterogeneous changes in astrocytic Ca^2+^ signals during Gap19 application, categorized as increased (D and E), decreased (F and G), or unchanged (H and I). Heatmaps depict the time‐course of Ca^2+^ signals in individual cells (D, F, and H), while quantification of normalized data reveals distinct response patterns (E, G, and I). Sample sizes: *n* = 70 cells from 6 mice (C), *n* = 39 cells (E), *n* = 19 cells (G), *n* = 12 cells (I). Significance levels: ****p < 0.0001 by Wilcoxon matched‐pairs signed rank test (E), two‐tailed paired *t* test (G and I).

The preceding findings demonstrate that inhibition of astrocytic Cx43 hemichannels enhanced respiratory output. To determine whether channel manipulation modulates astrocytic activation dynamics, we adapted established methodologies [42] to monitor Ca^2+^ signal fluctuations as a proxy for cellular activation. An AAV5‐GfaABC1D‐jGCaMP7b vector was delivered into the preBötC of C57BL/6J mice, followed by in vitro Ca^2+^ imaging in acute brainstem slices harvested four weeks postinjection. Slices were equilibrated in aCSF for at least 5 min prior to recording (Figure 7A,B). Bath application of Gap19 (1 μM) elicited three Ca^2+^ response subtypes across 70 imaged cells (Figure 7C). 55.7% (39/70) exhibited signal enhancement (sustained increase: 22 cells; transient increase: 17 cells; Figure 7D,E). Conversely, 27.1% (19/70) showed signal attenuation (Figure 7F,G), while 17.2% (12/70) displayed no detectable changes (Figure 7H,I). This functional heterogeneity in Ca^2+^ dynamics implicates Cx43 hemichannels in coordinating divergent activation states within preBötC astrocytes, revealing potential mechanisms for their pleiotropic roles in respiratory control.

### 3.7 Blockade of Astrocytic Cx43 Hemichannels Modulates preBötC^SST^ Neuron Activity

**FIGURE 8:**
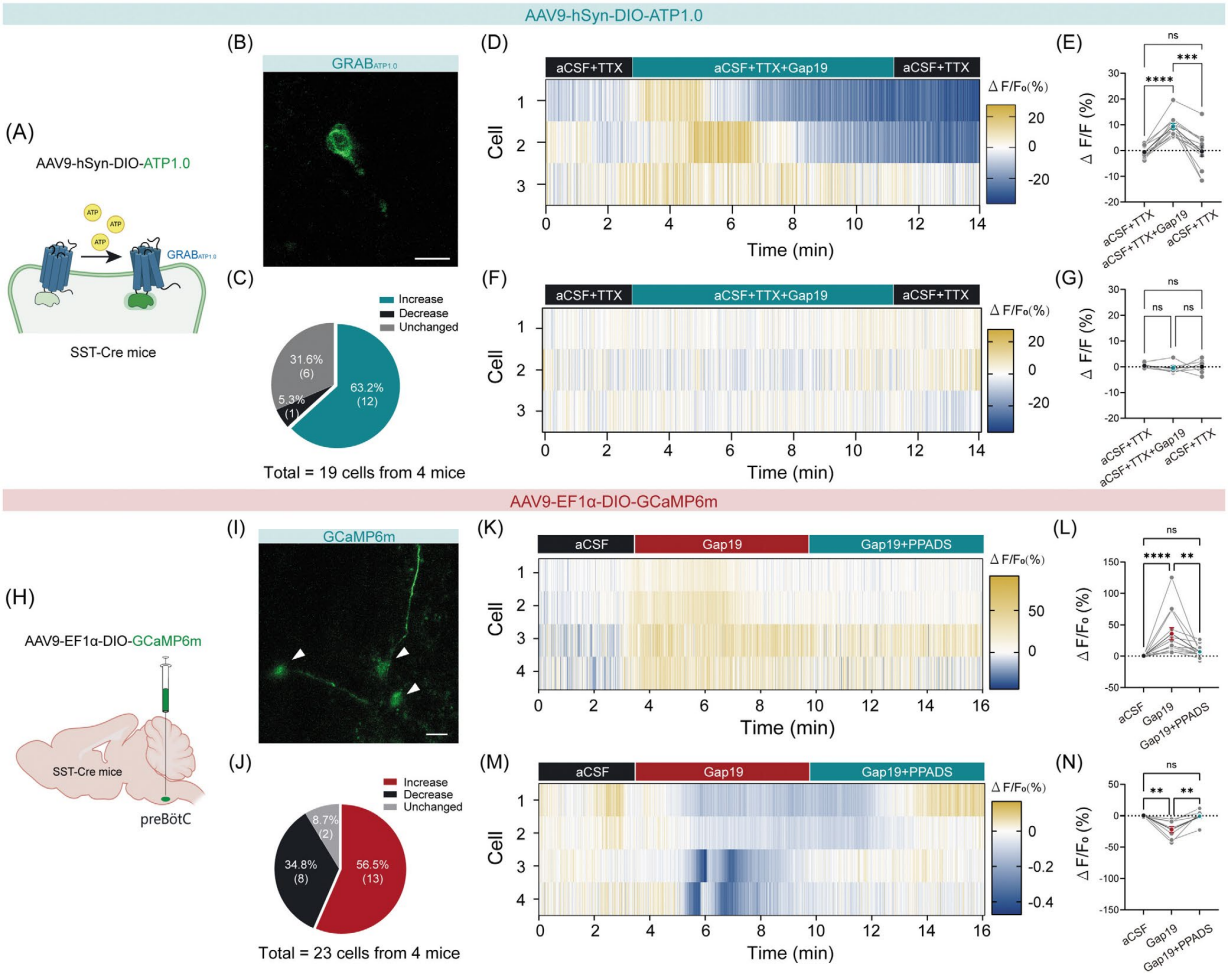
Blockade of astrocytic Cx43 hemichannels regulates preBötC^SST^ neuron activation. (A) Schematic of working principle of the AAV virus expressing an ATP sensor. (B) Typical confocal image depicting ATP accumulation around a neuron (green) in a slice from an SST‐Cre mouse with injection of a virus encoding ATP sensor into the preBötC. Scale bar, 10 μm. (C) Distinct response patterns of ATP signals around preBötC^SST^ neurons during bath application of Gap19 and TTX. (D–G) Under synaptic isolation by TTX administration (1 μM), bath application of Gap19 (1 μM) induced increased (D and E) and unchanged (F and G) ATP signals, as illustrated by heatmaps and quantification. (H) Schematic of the viral injection strategy. (I) Representative confocal image of GCaMP6m‐expressing neurons (green) in a slice from a GCaMP6m‐injected SST‐Cre mouse. White arrowheads show GCaMP6m‐expressing neurons. Scale bar, 20 μm. (J) Response patterns of preBötC^SST^ neurons during bath application of Gap19 (1 μM). (K–N) Heatmaps and line plots demonstrate that bath application of Gap19 induces increased (K and L) and decreased (M and N) Ca^2+^ signals of preBötC^SST^ neurons, an effect abolished by PPADS (1 μM) administration. Sample sizes: *n* = 19 cells from 4 mice (C), *n* = 12 cells (E), *n* = 6 cells (G), *n* = 23 cells from 4 mice (J), *n* = 13 cells (L), *n* = 8 cells (N). Significance levels: **p < 0.01, ***p < 0.001, ****p < 0.0001 by one‐way ANOVA with Tukey's multiple comparisons test (E, G, and N), one‐way ANOVA with Dunn's multiple comparisons test (L).

PreBötC^SST^ neurons are known to play a critical role in shaping respiratory output patterns [10]. We hypothesize that the blockade of astrocytic Cx43 hemichannels potentiates breathing through ATP‐mediated activation of preBötC^SST^ neurons. To validate this hypothesis, we injected a virus encoding an ATP sensor (AAV9‐hSyn‐DIO‐ATP1.0) into the preBötC of SST‐Cre mice (Figure 8A). This GPCR‐anchored fluorescent probe enables the detection of extracellular ATP dynamics [43]. Four weeks postinjection, ATP flux was monitored near preBötC^SST^ neurons in brainstem slices treated with TTX (1 μM) to block synaptic transmission. Bath application of Gap19 (1 μM) induced varying responses (*n* = 19 cells from 4 mice). Specifically, 63.2% of cells (*n* = 12/19) exhibited enhanced peri‐somatic fluorescence, 31.6% (*n* = 6/19) showed no significant change, and 5.3% (*n* = 1/19) exhibited decreased fluorescence (Figure 8B,C). Reversible fluorescence intensification was visualized in the heatmap (Figure 8D), with a mean increase of 9.4% that returned to baseline following washout (Figure 8E). The heatmap and statistical plot for neurons exhibiting no change in fluorescence intensity are also presented (Figure 8F,G). These results establish that the blockade of Cx43 hemichannels elevates ATP levels in the vicinity of approximately 60% of preBötC^SST^ neurons.

To further investigate the functional consequences of Cx43 hemichannel inhibition on preBötC^SST^ neuron activity, we performed in vitro Ca^2+^ imaging in brainstem slices. AAV9‐EF1α‐DIO‐GCaMP6m was injected into the preBötC of SST‐Cre mice (Figure 8H), enabling GCaMP6m expression in preBötC^SST^ neurons. Four weeks postinjection, Ca^2+^ dynamics were monitored in GCaMP6m‐expressing preBötC^SST^ neurons (*n* = 23 cells from 4 mice) (Figure 8I). Administration of Gap19 elicited three distinct neuronal response profiles (Figure 8J). Among the 23 recorded neurons, 56.5% (13/23) exhibited enhanced Ca^2+^ signals, an effect that was attenuated by the P2 receptor antagonist PPADS (Figure 8K,L). By contrast, 34.8% (8/23) displayed suppressed Ca^2+^ activity, which was also reversed by the application of PPADS (Figure 8M,N). The remaining 8.7% (2/23) showed no significant changes. These findings suggest that blockade of Cx43 hemichannels of a subpopulation of preBötC astrocytes induces an increase in ATP release, which subsequently activates P2 receptors to elevate preBötC^SST^ neuronal excitability.

### 3.8 P2Y1 Receptors in the preBötC Mediate Cx43 Hemichannel Blockade‐Induced Enhanced Respiratory Drive

**FIGURE 9:**
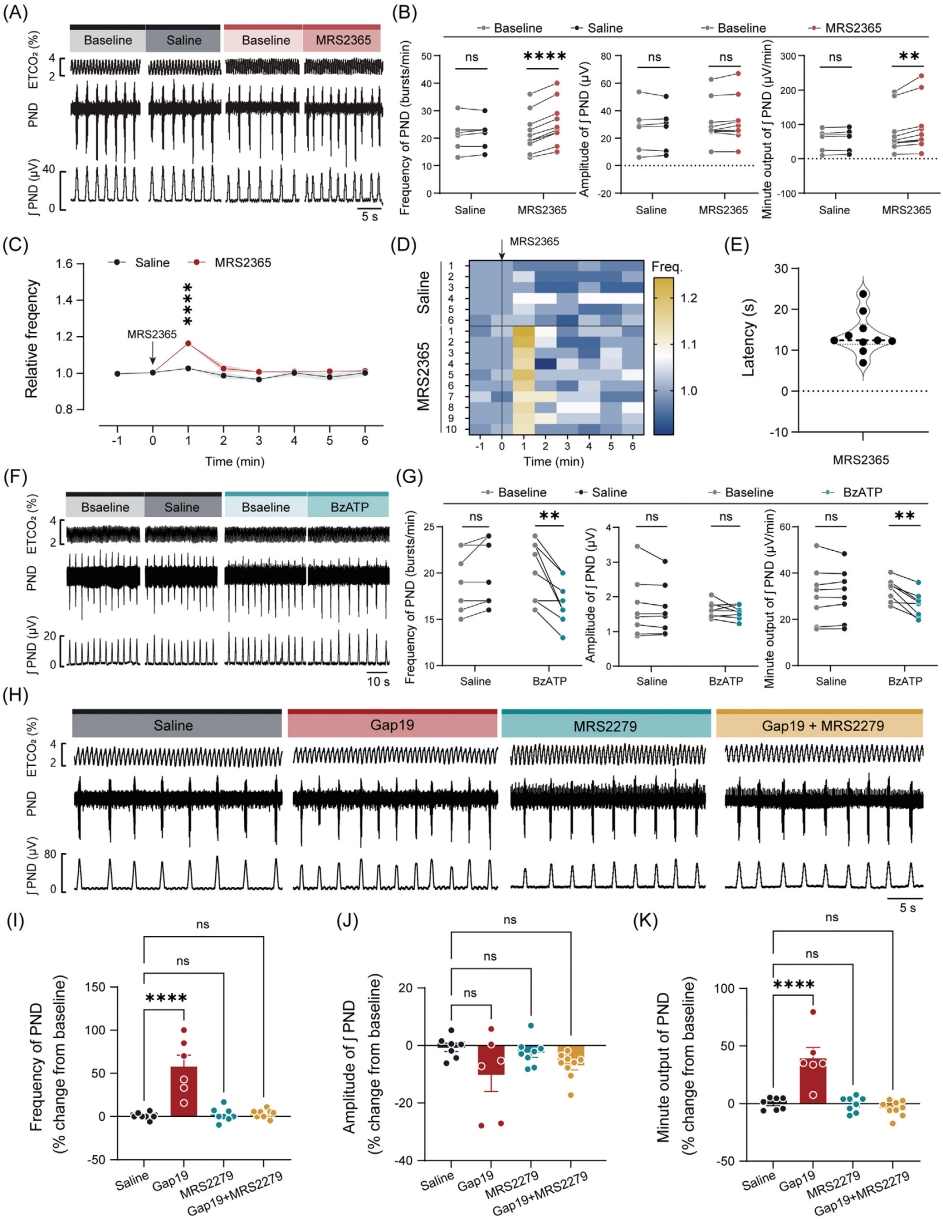
Blockade of P2Y1 receptors suppresses the enhanced PND induced by Gap19. (A) Representative traces illustrating the effects of injections of saline and MRS2365 (100 μM, 80 nL per injection) into the preBötC of C57BL/6J mice on PND activity. (B) Quantitative analysis of PND parameters. (C) Time course of the effect of the injection of saline and MRS2365 on average PND frequency. (D) Heatmap showing the time‐course changes in PND frequency for individual mice during saline and MRS2365 administration. (E) Quantification of the response latency following MRS2365 injection. (F) Representative traces showing the effect of injections of saline and BzATP (100 μM, 80 nL per injection) on PND activity. (G) Quantitative analysis of PND activity. (H) Representative traces showing that the injection of Gap19, but not saline, induced an increase in PND frequency, an effect that was eliminated by co‐administration of MRS2279 (100 μM, 80 nL per injection). (I–K) Quantification of normalized PND frequency, amplitude, and minute output in response to the injection of either saline or drugs in the preBötC. Sample sizes: *n* = 6 mice for saline (B–D), *n* = 10 mice for MRS2365 (B–E), *n* = 8 injections from 5 mice for saline (G), *n* = 8 injections from 5 mice for BzATP (G), *n* = 7 injections from 5 mice for saline (I–K), *n* = 6 mice for Gap19 (I–K), *n* = 8 injections from 5 mice for MRS2279 (I–K), *n* = 9 injections from 5 mice for MRS2279 plus Gap19 (I–K). Significance levels: **p < 0.01, ****p < 0.0001 by two‐tailed paired *t*‐test (B and G), two‐way ANOVA with Bonferroni's multiple comparisons test (C), one‐way ANOVA with Dunnett's multiple comparisons test (I–K).

To identify the specific purinergic receptor subtypes mediating the effects of Cx43 hemichannel blockade, we injected the P2Y1 agonist MRS2365 (100 μM, 80 nL per injection) into the preBötC from anesthetized C57BL/6J mice. Notably, administration of this drug significantly increased PND frequency and minute output but did not affect amplitude (Figure 9A–D), with an average latency‐to‐onset of 13.8 s (Figure 9E). In contrast, injection of the selective P2X receptor agonist BzATP (100 μM, 80 nL per injection) into the preBötC decreased PND frequency and minute output (Figure 9F,G). These findings suggest that the activation of preBötC^SST^ neurons following Cx43 hemichannel blockade is mediated by ATP acting on P2Y1 receptors rather than P2X receptors.

To further elucidate the role of P2Y1 receptors in the preBötC in mediating the effects of Cx43 hemichannel blockade on central respiratory drive, we injected saline, Gap19, MRS2279 (a selective and high‐affinity P2Y1 receptor antagonist), or a combination of MRS2279 and Gap19 directly into the preBötC and subsequently assessed the changes in PND activity. We demonstrated that injection of either saline or MRS2279 (100 μM, *n* = 8 injections in 5 mice) alone did not significantly alter PND activity. In contrast, administration of Gap19 alone significantly increased both PND frequency and minute output, indicating enhanced central respiratory drive. Importantly, concurrent injection of MRS2279 and Gap19 (*n* = 9 injections in 5 mice) abolished the effects of Gap19 on PND parameters (Figure 9H–K). These results collectively demonstrate that P2Y1 receptors are essential for mediating the enhanced respiratory drive induced by Cx43 hemichannel blockade in the preBötC, highlighting the critical role of P2Y1 receptor signaling in respiratory pattern regulation.

## 4 Discussion

Respiratory rhythmogenesis and pattern stabilization are essential for maintaining arterial blood gas homeostasis and metabolic equilibrium. This regulatory process critically involves the ventrolateral medullary respiratory network, particularly through the integrative functions of the preBötC. In this study, we establish that preBötC astrocytes stabilize breathing patterns via Cx43 channels and purinergic signaling pathways. Key findings demonstrate that patterned astrocytic activation is temporally coupled to sighs and TAB events. Photostimulation of preBötC astrocytes potentiates respiratory drive in anesthetized mice, an effect replicated by pharmacological inhibition of Cx43 hemichannels. Moreover, genetic knockdown of astrocytic *Gja1* significantly enhances resting ventilation. Cx43 hemichannel blockade elevates activation levels of preBötC^SST^ neurons via astrocyte‐derived ATP activation of neuronal purinergic receptors, an effect antagonized by selective P2Y1 receptor inhibition. Collectively, we identify an astrocyte‐to‐neuron regulatory axis: Cx43 hemichannel‐dependent ATP release → purinergic signaling → preBötC^SST^ neuron activation → respiratory motor output, highlighting the critical role of astrocytic Cx43 hemichannels in stabilizing breathing patterns.

### 4.1 Astrocytic Regulation of Respiratory Motor Output

Respiratory homeostasis critically depends on dynamic neuro‐astrocytic interactions. Astrocytes modulate breathing via actions on rCPG and central respiratory chemoreceptors. Nucleus tractus solitarii (NTS) astrocytes regulate respiratory output through glutamatergic signaling [44], modulation of TRPV1 channels [45], contributing to respiratory control in OSA [46] and Alzheimer's disease models [47]. Retrotrapezoid nucleus (RTN) astrocytes serve as central respiratory chemoreceptors providing excitatory drive to breathing [48], modulating neuronal chemosensitivity and hypercapnic ventilatory responses through Na^+^‐HCO_3_^−^ cotransporter [49], Kir4.1 channels [20] and prostaglandin E2 [50]. PreBötC astrocytes modulate rhythmogenesis, breathing pattern [14, 16] and hypoxic ventilatory responses [15]. Building on these accumulated data, we demonstrate herein that using in vivo fiber photometry in freely behaving mice, we reveal event‐locked astrocytic activation: preBötC Ca^2+^ transients preferentially phase‐lock to sighs and TAB events, not eupnea, suggesting context‐specific pattern modulation. Okada et al. identified a subset of preBötC astrocytes exhibiting Ca^2+^ oscillations preceding inspiratory neuron activity in brainstem slices [51], potentially reflecting slice‐specific phenomena or distinct subpopulations. Gliotransmitter release following Ca^2+^ influx may modulate neuronal excitability and rhythmogenesis [52]. On the other hand, the enhanced astrocytic activation could also be a consequence of the amplified respiratory events, as the changes in neuronal activity during these events may lead to the activation of molecular targets located on astrocytes and the subsequent increase in Ca^2+^ levels [53, 54]. However, Schnell et al. did not detect periodic Ca^2+^ rises in astrocytes coupled with neuronal rhythmic activities in the preBötC of rhythmic slice preparations from neonatal mice [55]. Hence, they suggested that astrocytes exhibit respiratory‐rhythmic Ca^2+^ fluctuations only when they fail to prevent synaptic glutamate accumulation, in the case when astrocyte glutamate transport is suppressed or neuronal discharge is excessive. Collectively, these findings establish preBötC astrocytes as context‐selective regulators whose temporal activation patterns are tuned to specific respiratory patterns.

Furthermore, our findings demonstrate that photostimulation of preBötC astrocytes significantly enhances respiratory drive, as evidenced by a marked increase in PND frequency. This aligns with the reports that photostimulation of preBötC astrocytes induces inspiratory neuronal firings [51], as well as chemogenetic studies showing enhanced resting breathing rates, sigh frequency, and rhythm variability [14]. While accumulating evidence supports the notion that photostimulation of astrocytes elicits diverse physiological effects [56, 57, 58], this approach remains subject to debate. Notably, ChR2 and its variants function as nonselective cation channels, permitting the movement of Na^+^, K^+^, H^+^, and Ca^2+^ across cellular membranes [48, 59]. A recent study reveals that photostimulation of ChR2‐expressing astrocytes elevated extracellular K^+^ concentration [60], raising concerns about potential off‐target effects. To address this issue in the present study, we quantified cFos immunoreactivity in both astrocytes and neurons within the preBötC following programmed photostimulation. Our results revealed a significant increase in cFos^+^ astrocytes and neurons in both the ipsilateral and contralateral preBötC, thereby confirming the efficacy of photostimulation in activating astrocytes.

### 4.2 Regulation of Astrocytic Activation by Cx43 Hemichannels

Neuro‐astrocytic signaling involves diverse mechanisms, including gap junction‐mediated network integration. Astrocytes form functional syncytia through extensively coupled gap junction networks [61, 62, 63]. These intercellular channels comprise connexin proteins, with 21 isoforms identified in humans [64]. Among these, astrocytic Cx43 channels demonstrate the broadest tissue distribution across mammalian systems, particularly within cardiovascular and neural tissues [65]. Based on the findings from RNA sequencing, RNAscope‐FISH, and qPCR analyses, we identify Cx43 as the predominant connexin protein expressed in preBötC astrocytes. Additionally, Cx30 channels are densely and specifically localized in preBötC astrocytes, suggesting a potential role in the regulation of breathing. However, the knockdown of Cx43 channels did not alter the expression levels of Cx30 channels through a compensatory mechanism as reported previously [40, 41], indicating that Cx30 channels may not be directly involved in the acute modulation of respiratory control. Despite this, our current results do not entirely rule out the possibility of Cx30 channels contributing to the maintenance of respiratory homeostasis. Further investigations are warranted to fully elucidate the functional significance of Cx30 channels in respiratory regulation.

We demonstrate that both pharmacological blockade and knockdown of astrocytic Cx43 channels significantly enhance respiratory drive and motor output, as evidenced by increased PND, elevated BF, and MV. These results underscore the essential role of Cx43 channels in maintaining breathing pattern stability. To investigate the functional consequences of Cx43 channel disruption, we employed distinct experimental approaches: unilateral pharmacological blockade and bilateral genetic knockdown. Unilateral injection of CBX or Gap19 into the preBötC was used to achieve acute, localized disruption of Cx43 channel function, while the contralateral side served as an internal control within the same animal. Given that preBötC neurons project to the contralateral preBötC [39], it is plausible that bilateral compensatory mechanisms exist to maintain respiratory homeostasis. Consequently, unilateral knockdown effects might be masked by contralateral compensation, obscuring the full respiratory phenotype. To eliminate such compensatory effects and ensure a robust and unambiguous functional readout, bilateral AAV‐mediated knockdown of Cx43 channels was necessary. This approach provided a comprehensive assessment of the role of Cx43 channels in respiratory regulation, free from confounding compensatory influences.

Using Gap19, a selective Cx43 hemichannel inhibitor that preserves gap junctional communication [66], we implicate hemichannel‐mediated paracrine signaling (e.g., ATP/glutamate release) in respiratory modulation. Gap19 application elicited a tripartite Ca^2+^ response profile in preBötC astrocytes, with enhanced activation observed in 58.6% of cells. This functional heterogeneity underscores both the regulatory role of constitutive Cx43 hemichannel activity and the operational diversity of preBötC astrocytes. Notably, the mechanisms driving Gap19‐induced Ca^2+^ dynamics remain unresolved.

Previous studies show that Cx43 hemichannels serve as pathways for Ca^2+^ influx [67, 68, 69]. Hemichannel blockade typically reduces intracellular Ca^2+^, aligning with our observed astrocytic inhibition subset (27.1%). We hypothesize two plausible mechanisms for Gap19‐induced Ca^2+^ elevations: mitochondrial Ca^2+^ buffering [70] and ATP‐dependent endoplasmic reticulum Ca^2+^ handling [71]. Additionally, the secondary pathways of Gap19, like alterations in the astrocytic extracellular environment causing increased intracellular calcium ion concentration via various pathways, may also be important factors [72, 73]. Future studies must delineate the mechanistic basis of this differential astrocytic activation.

### 4.3 Purinergic Signaling Through P2Y1 Receptors Activates preBötC^SST^ Neurons

In this study, we reveal that blockade of Cx43 hemichannels induced astrocytic activation, subsequent ATP release, acting on preBötC^SST^ neurons. Under synaptic isolation, Cx43 hemichannel inhibition significantly increased pericellular ATP concentrations surrounding ∼60% of preBötC^SST^ neurons. This localized accumulation originates from astrocytic activation following Cx43 hemichannel blockade. Astrocytic ATP is released through connexin and pannexin channels, or vesicular exocytosis, in turn allowing rapid and spatially precise delivery of ATP to specific synaptic targets, as reported previously [74, 75]. While recent studies have shown that inhibition of astrocytic Cx43 hemichannels reduces ATP release [76, 77], our findings reveal increased ATP accumulation following Cx43 hemichannels blockade. We hypothesize that this discrepancy may be explained by alternative mechanisms, such as pannexin channels or vesicular exocytosis.

We demonstrate that the purinergic signaling in the preBötC was mediated by P2Y1 receptors [78], in line with another study demonstrating that activation of preBötC P2Y1 receptors increases ventilation [15]. In the preBötC, 87% of SST^+^ neurons are glutamatergic neurons that convey excitatory signals from the preBötC to shape respiratory motor output [10, 79, 80, 81]. Our results demonstrate that blockade of Cx43 hemichannels significantly amplified Ca^2+^ signaling in preBötC^SST^ neurons and elevated ATP concentration in their surrounding environment. Additionally, blockade of Cx43 hemichannels induces heterogeneous activation levels of preBötC^SST^ neurons, with approximately 60% of neurons exhibiting increased activity. We injected the P2Y1 receptor agonist into the preBötC and induced a significant enhancement in the central respiratory drive, mirroring the effects observed following acute Cx43 hemichannels blockade. However, when P2Y1 receptors were pharmacologically blocked, the enhancement in central respiratory drive was no longer observed, suggesting that this effect was mediated by ATP acting on P2Y1 receptors. Collectively, these findings establish that P2Y1 receptors are critical for ATP‐mediated activation of preBötC^SST^ neurons. Purinergic receptors in the preBötC are not exclusive to SST neurons, but are broadly expressed across multiple cell types, including excitatory neurons, inhibitory interneurons, and glial cells themselves. Studies have shown that ATP can modulate the excitability of preBötC neurons via purinergic receptors and contribute to respiratory regulation under hypoxic stress [15, 82]. Thus, purinergic signaling targets multiple cell populations and is not restricted to SST^+^ neurons. Regrettably, the present study did not systematically resolve the activation profiles of individual neuronal subtypes.

## 5 Conclusions

Collectively, we demonstrate that both pharmacological blockade and genetic knockdown of astrocytic Cx43 channels in the preBötC induce a high‐frequency breathing pattern, thereby enhancing respiratory motor output. This effect is most likely mediated by astrocytic activation and subsequent ATP release, which activates P2Y1 receptors on preBötC^SST^ neurons. We uncover a novel mechanism by which astrocytic Cx43 channels in the preBötC play an essential role in stabilizing breathing patterns. These findings identify potential molecular targets for the central treatment of hyperventilation‐associated disorders.

## Author Contributions

Study design and manuscript drafting/revising: S.W., F.Y., and L.S.; conducting the experiments, acquisition of the data, and figure drawing: X.Z., H.Y., X.J., and Y.L.; acquisition of the data and statistical analysis: X.Z., Y.C., X.W., K.Z., X.Z., and T.D. All authors have read and approved the final version of the manuscript.

## Ethics Statement

All experiments were performed in accordance with the Guide for the Care and Use of Laboratory Animals and were approved by the Animal Care and Ethical Committee of Hebei Medical University (Hebmu‐P2023052).

## Conflicts of Interest

The authors declare no conflicts of interest.

## Supporting information


**Data S1:** cns70668‐sup‐0001‐DataS1.xlsx.

**Figure S1–S3:** cns70668‐sup‐0002‐Figures.docx.

## Data Availability

The data that support the findings of this study are available from the corresponding author upon reasonable request.
