# Supplementary material for: Astrocytic Connexin43 Channels Are Essential for Breathing Pattern Stabilization in the preBötzinger Complex
Source: CNS Neurosci Ther. 2025 Nov 24;31(11):e70668. doi: 10.1111/cns.70668 (PMC12641571; doi:10.1111/cns.70668)
Supplement: Supplementary file 2 — Figure S1–S3: cns70668‐sup‐0002‐Figures.docx. [file CNS-31-e70668-s002.docx]

**Supplementary Figures**

**
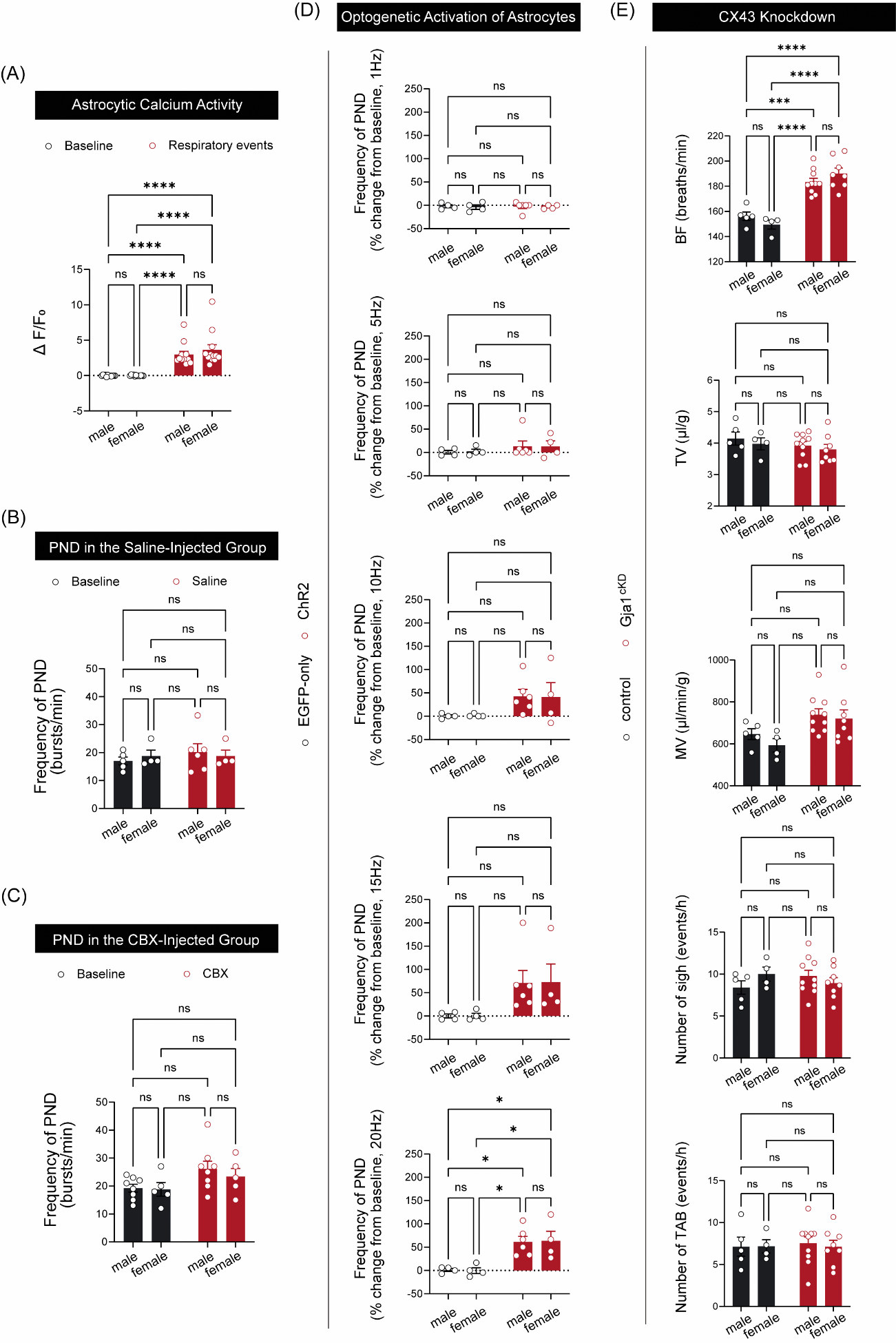
**

**Supplementary Figure 1 Analysis of sex difference in respiratory control.** (A) Quantitative analysis of Ca^2+^ signals changes during baseline and respiratory events. ΔF/F_0_ (%) indicates the change in signal fluorescence normalized to baseline fluorescence (from the median of the entire time series). (B) Quantitative analysis of PND activity in saline-injected mice. (C) Quantitative analysis of PND activity in CBX-injected mice. (D) Quantification of normalized PND frequency during optogenetic activation of astrocytes in the preBötC. (E) Quantitative analysis of BF, TV, MV, sighs and TAB events over a 1-hour period. Significance levels: *P* > 0.05, ^*^*P* < 0.05, ^***^*P* < 0.001, ^****^*P* < 0.0001 by Tukey's multiple comparisons test (A-E). ns, not significant.


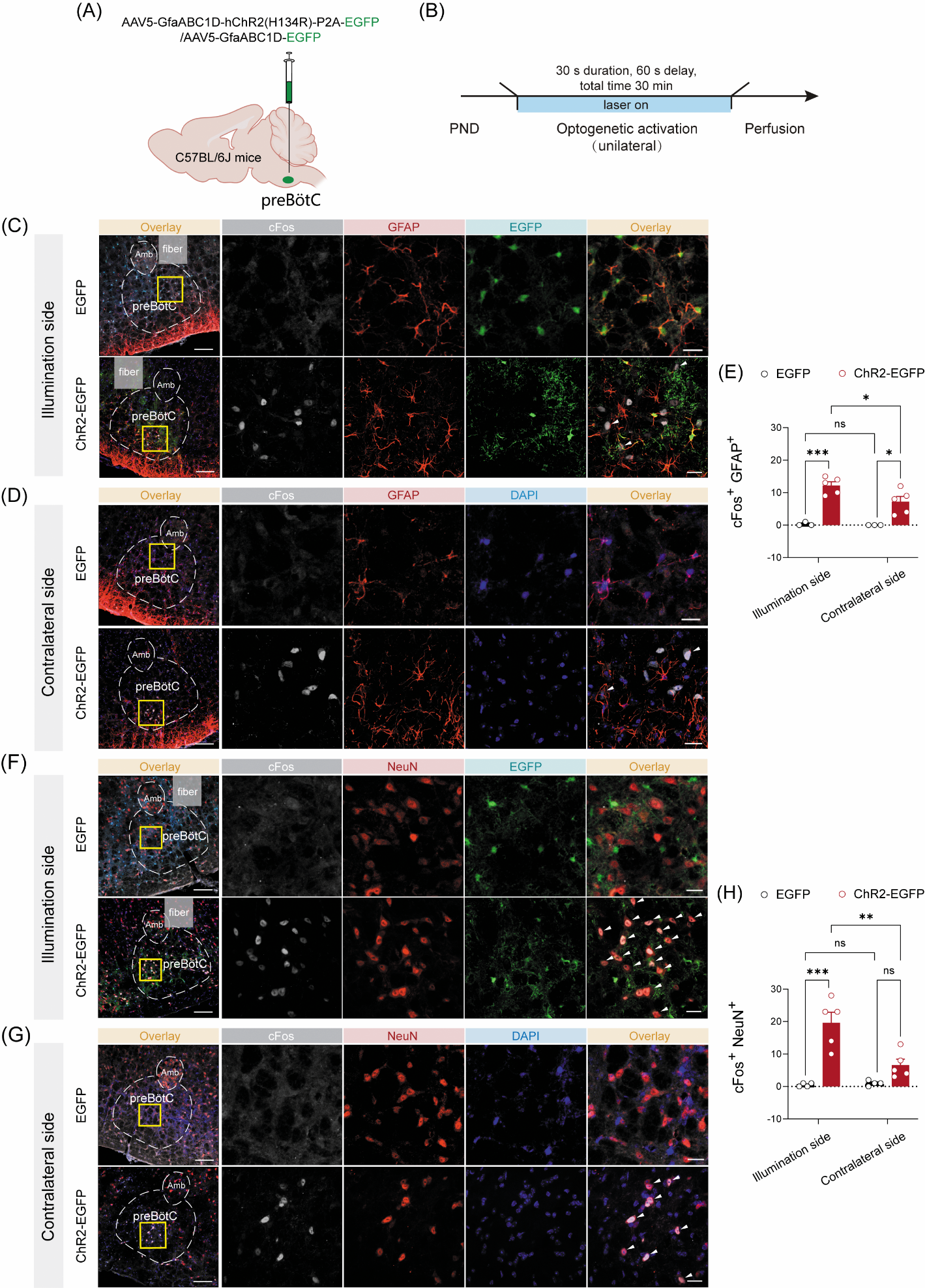


**Supplementary Figure 2 Unilateral illumination of the preBötC activated bilateral astrocytes and neurons.** (A) Schematic diagram showing viral injection strategy for optogenetics. (B) Experimental protocol for unilateral illumination of the preBötC using optogenetic stimulation. (C) Unilateral illumination enhanced ipsilateral astrocytic activation. The left images show an overlay image of cFos (gray), GFAP (red) and EGFP (green) in the preBötC of C57BL/6J mice injected with virus expressing EGFP (top) and ChR2-EGFP (bottom). Scale bars: 100 μm. The right panels are magnified views of the yellow boxed region, with white arrowheads indicating triple labeling of cFos, GFAP, and EGFP. Scale bars: 20 μm. (D) Unilateral illumination of the preBötC enhanced contralateral astrocytic activation. The left images display cFos (gray), GFAP (red) and nuclear counterstain DAPI (blue) in the contralateral preBötC. Scale bars: 100 μm. The right panels are magnified views of the yellow boxed region, with white arrowheads indicating double labeling of cFos and GFAP. Scale bars: 20 μm. (E) Quantitative analysis of cFos^+^ and GFAP^+^ co-labeled cells. (F) Unilateral illumination of the preBötC enhanced ipsilateral neuronal activation. The left image displays cFos (gray), NeuN (red) and EGFP (green) in the preBötC of C57BL/6J mice injected with virus expressing EGFP and ChR2-EGFP. Scale bars: 100 μm. The right panels are enlarged views of the yellow box region highlighted in the left image. White arrowheads indicate double labeling for cFos and NeuN. Scale bars: 20 μm. (G) Unilateral illumination of the preBötC enhanced contralateral neuronal activation. The left image displays cFos (gray), NeuN (red) and nuclear counterstain DAPI (blue) within the contralateral preBötC of C57BL/6J mice. Scale bars: 100 μm. The right panels are enlarged views of the yellow box region highlighted in the left image. Scale bars: 20 μm. (H) Quantitative analysis of cFos^+^ and NeuN^+^ co-labeled cells. All images were acquired at a bregma level of −6.95 mm. Sample sizes: n = 3 mice in group EGFP (E), n = 5 mice in group ChR2-EGFP (E), n = 4 mice in group EGFP (H), n = 5 mice in group ChR2-EGFP (H). Significance levels: ^**^*P* < 0.01, ^****^*P* < 0.0001 by two-way ANOVA with Tukey's multiple comparisons tests (E, H). ns, not significant.


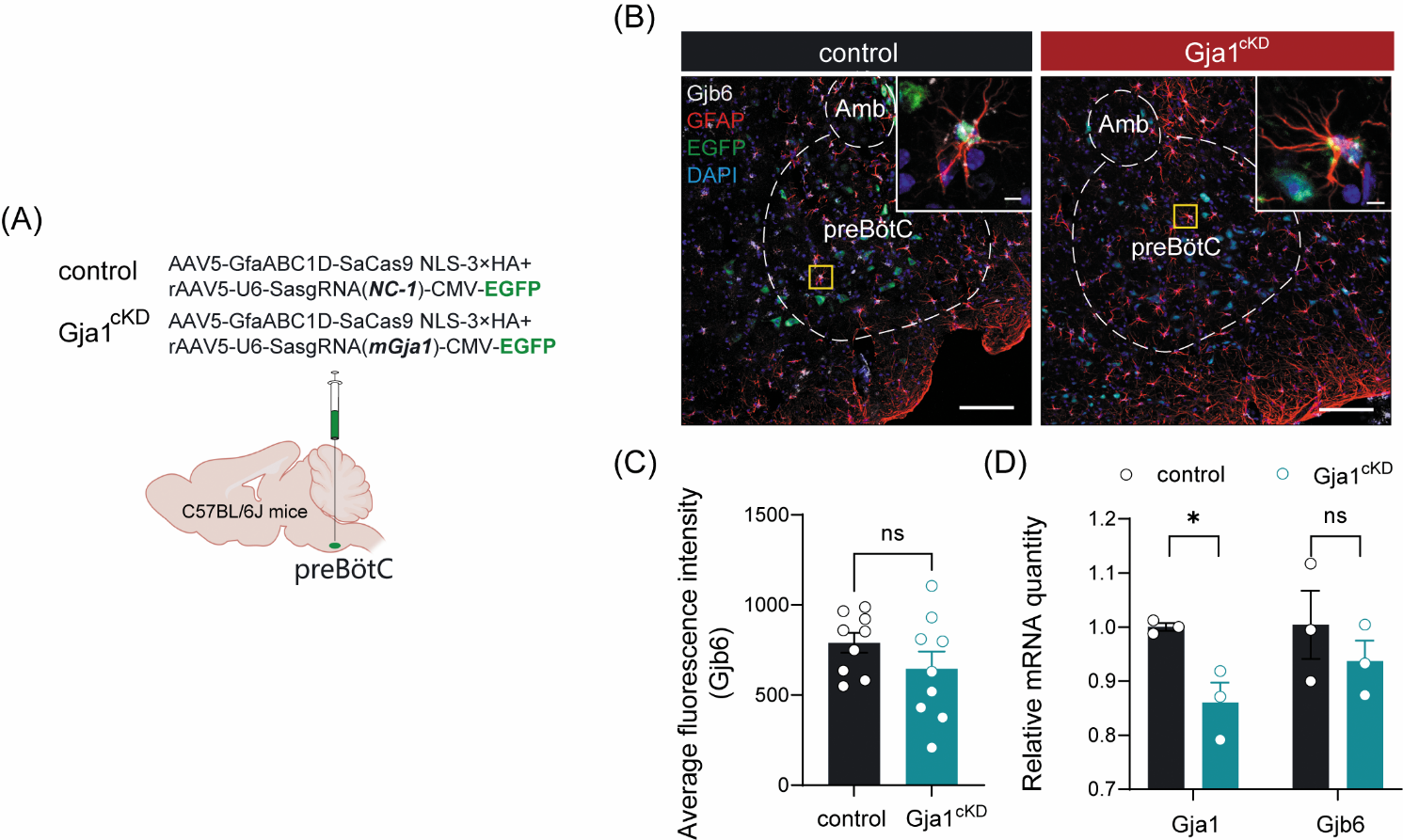


**Supplementary Figure 3 Expression levels of Gjb6 following Gja1 knockdown.** (A) Schematic diagram showing the viral injection strategy. (B) RNAscope-FISH and immunohistochemical assessment of *Gjb6* mRNA levels. Notably, expression level of *Gjb6* mRNA was essentially unchanged in Gja1^cKD^ mice (right) relative to controls (left). Scale bar: 100 μm. The inserts indicate enlarged views derived from the squared regions. Scale bar: 5 μm. (C) Average fluorescence intensity (normalized to area). (D) Quantitative analysis using qPCR demonstrates that expression levels of Gjb6 mRNA in Gja1^cKD^ mice showed no significant change compared to control mice. Sample sizes: n = 9 mice in each group (C), n = 3 mice in each group (D). Significance levels: ^*^*P* < 0.05 by two-tailed unpaired *t* test (C, D). ns, not significant.
